# A new dystrophin-deficient rat model mirroring exon skipping in patients with *DMD* exon 45 deletions

**DOI:** 10.1242/dmm.052578

**Published:** 2026-02-04

**Authors:** Tao Wang, Cynthia Daoud, Auriane Dubois, Guillaume Corre, Jessica Bellec, Matteo Bovolenta, Louise Philidet, Alan Dorval, Nathalie Bourg, Carinne Roudaut, Sonia Albini, Ganesh Warthi, Abbass Jaber, Isabelle Richard

**Affiliations:** ^1^ Généthon, 91000 Evry France; ^2^ Université Paris-Saclay, Univ. Evry, INSERM, Généthon, INTEGRARE Research Unit UMR_S951, 91000, Evry-Courcouronnes, France

**Keywords:** Duchenne muscular dystrophy, Rat, Exon skipping, Cardiomyopathy

## Abstract

Pathogenic variants in the dystrophin (*DMD*) gene cause muscle-wasting disorders ranging from the milder Becker muscular dystrophy (BMD) to the more severe Duchenne muscular dystrophy (DMD). Exon 45 deletion is the most-frequent single-exon deletion in patients diagnosed with DMD. Here, we generated a novel rat model with an exon 45 deletion using CRISPR/Cas9. The *Dmd^Δ45^* rat recapitulate key features of DMD, including progressive skeletal muscle degeneration, impaired muscle and cardiac function, and cognitive deficits. Transcriptomics analyses revealed gene expression patterns consistent with dystrophin deficiency. In skeletal muscle, we observed a transition from early stress responses and regeneration to chronic inflammation, fibrosis and metabolic dysfunction. Cardiac profiles similarly progressed from early inflammatory responses to fibrotic remodelling and metabolic impairment. Notably, *Dmd*^Δ45^ rats displayed a milder phenotype than other DMD rat models. This attenuation is likely due to spontaneous exon skipping, particularly of exon 44, which partially restores the reading frame and increases revertant dystrophin-positive fibres with age. Downregulation of spliceosome-related genes suggests a potential mechanism for this exon skipping. Overall, this model provides valuable insights into phenotypic variability and therapeutic exon-skipping strategies.

## Teaser

**Summary:** A new rat model of Duchenne muscular dystrophy with exon 45 deletion reveals progressive muscle and heart disease, while uncovering natural exon skipping that attenuates disease severity.

## INTRODUCTION

Duchenne muscular dystrophy (DMD) is a severe X-linked recessive muscular disorder caused by pathogenic variants in the *DMD* gene, which encodes dystrophin, a structural protein critical for muscle fibre integrity (Hoffman et al., 1987; Koenig et al., 1987). With an incidence of approximately 1 in 5000 male births, DMD is the most common and devastating form of childhood muscular dystrophy (Duan et al., 2021; Moat et al., 2013). Variants that do not disturb the reading frame in the same gene can cause Becker muscular dystrophy (BMD), a clinically milder form characterized by the expression of partially functional dystrophin forms, typically characterized by later onset and slower disease progression (Mercuri et al., 2019; Davies and Vogt, 2024).

The protein product of *DMD*, dystrophin, is predominantly expressed in skeletal and cardiac muscle tissues. It is a sub-sarcolemmal protein with several binding domains, that forms a mechanical bridge between the extracellular matrix (ECM) and the cytoskeleton. Dystrophin is a pivotal constituent of the dystrophin-associated glycoprotein complex (DAGC) (Ervasti and Campbell, 1991), crucial not only for maintaining muscle fibre rigidity (Garcia-Pelagio et al., 2011) but also for shielding the muscle against mechanical stress encountered during contractions (Blaauw et al., 2010; Petrof et al., 1993). Its deficiency leads to perturbation of the assembly of the DAGC and destabilization of the muscle membrane, making it more fragile and sensitive to mechanical stress (Ervasti, 2007; Petrof, 1998).

Clinically, DMD manifests early, usually under the age of 2−3 years, with proximal muscle weakness – particularly in the lower limbs – accompanied by elevated serum levels of muscle creatine kinase CKM (hereafter referred to as CK) levels as a result of ongoing muscle fibre damage (Duan et al., 2021). Muscle weakness progresses rapidly, leading to difficulties standing up and an inability to walk by the age of 10 to 12 years. Respiratory muscles become affected, necessitating assisted ventilation at around the age of 20. While motor deficits dominate the early clinical picture, cognitive impairments, especially in working memory and executive function, are also frequently reported (Battini et al., 2018), although these typically remain stable over time. Cardiac and respiratory complications generally emerge during the second or third decade of life and are now recognized as the leading causes of mortality in DMD (Schultz et al., 2022; Duan, 2018).

The *DMD* gene is the largest known protein-coding gene, spanning over 2.6 million base pairs and comprising 79 exons. Owing to its size, it is highly susceptible to mutation, with thousands of pathogenic variants identified in patients diagnosed with DMD and BMD (Aartsma-Rus et al., 2006). In DMD, these variants typically result in the absence of functional dystrophin protein, with ∼60–70% being exon deletions and ∼20% comprising point mutations, small insertions or deletions (Tuffery-Giraud et al., 2009; Aartsma-Rus et al., 2006; Magri et al., 2011). By contrast, BMD variants tend to preserve the open reading frame, allowing the production of truncated but partially functional dystrophins. Mutation hotspots have been identified in the *DMD* gene, notably in exons 3–9 and exons 45–55 (Nakamura, 2017; Nakamura et al., 2016), with the latter accounting for nearly half of all deletions observed in patients diagnosed with DMD worldwide (Tuffery-Giraud et al., 2009; Aartsma-Rus et al., 2006; Cunniff et al., 2009; Takeshima et al., 2010).

Current DMD care relies on an early, multidisciplinary management focused on symptom relief with, especially, the use of glucocorticoids to slow disease progression. In parallel, therapeutic strategies aimed at restoring dystrophin expression have shown promise, with some gaining regulatory approval. These include exon skipping by using antisense oligonucleotides (AONs) to bypass mutated exons during mRNA splicing (Niks and Aartsma-Rus, 2017). Another promising, mutation-independent approach involves the delivery of shortened dystrophin constructs (micro-dystrophins) by adeno-associated viral (AAV) vectors (Bengtsson et al., 2025).

The exon 45–55 region is of particular interest for exon skipping therapies, as restoring the reading frame here can yield a BMD-like dystrophin and milder clinical phenotype. Exon 45 skipping, in particular, has shown therapeutic potential (Young et al., 2017; Wagner et al., 2021), and is the basis for the FDA-approved AON casimersen (Torres-Masjoan et al., 2025). This underscores the need for disease models that replicate this hotspot for the development and testing of AONs, and gene editing tools. While the *mdx* mouse model, harbouring a point mutation in exon 23, is widely used, its mild phenotype and limited cardiac involvement reduce its translational relevance (Hammers et al., 2020). By contrast, DMD rat models, enabled by recent advances in genome-editing technologies, exhibit more-severe and human-like disease progression, including pronounced muscle degeneration, fibrosis and cardiac involvement (Taglietti et al., 2022; Nakamura et al., 2014; Larcher et al., 2014; Krishnan et al., 2020). To date, these include the *Dmd^mdx^* rat with an exon 23 deletion (Larcher et al., 2014), the *Dmd^Δ3–16^* rat (Nakamura et al., 2014) and the *Dmd^Δ52^* rat carrying a deletion of exon 52 (Taglietti et al., 2022). However, no rat model has yet addressed the clinically relevant exon 45 hotspot. To fill this gap, we generated a novel *Dmd^Δ45^* rat model featuring a targeted deletion of exon 45. This model exhibited hallmark features of DMD, including progressive impairments in skeletal muscle, cardiac function and cognitive performance, alongside elevated muscle damage biomarkers, impaired muscle function and overall reduced lifespan. Interestingly, despite these features, the phenotype in this model was less severe compared to other reported DMD rat models. This attenuation may be attributed in part to preferential progressive skipping of exon 44, which restores the reading frame and results in the expression of partially functional dystrophin in the form of revertant fibres. Transcriptomic analysis revealed age-dependent dysregulation of genes involved in fibrosis and the spliceosome, shedding light on disease mechanisms and the biological basis of spontaneous exon skipping. Collectively, the *Dmd^Δ45^* rat represents a valuable model for dissecting the pathophysiology of DMD/BMD associated with exon 45–55 pathogenic variants, and offers a robust preclinical platform for the development and testing of exon-skipping and gene-editing therapies targeting this critical mutational hotspot.

## RESULTS

### Generation and validation of the *Dmd*^Δ*45*^ rat model

**Fig. 1.**
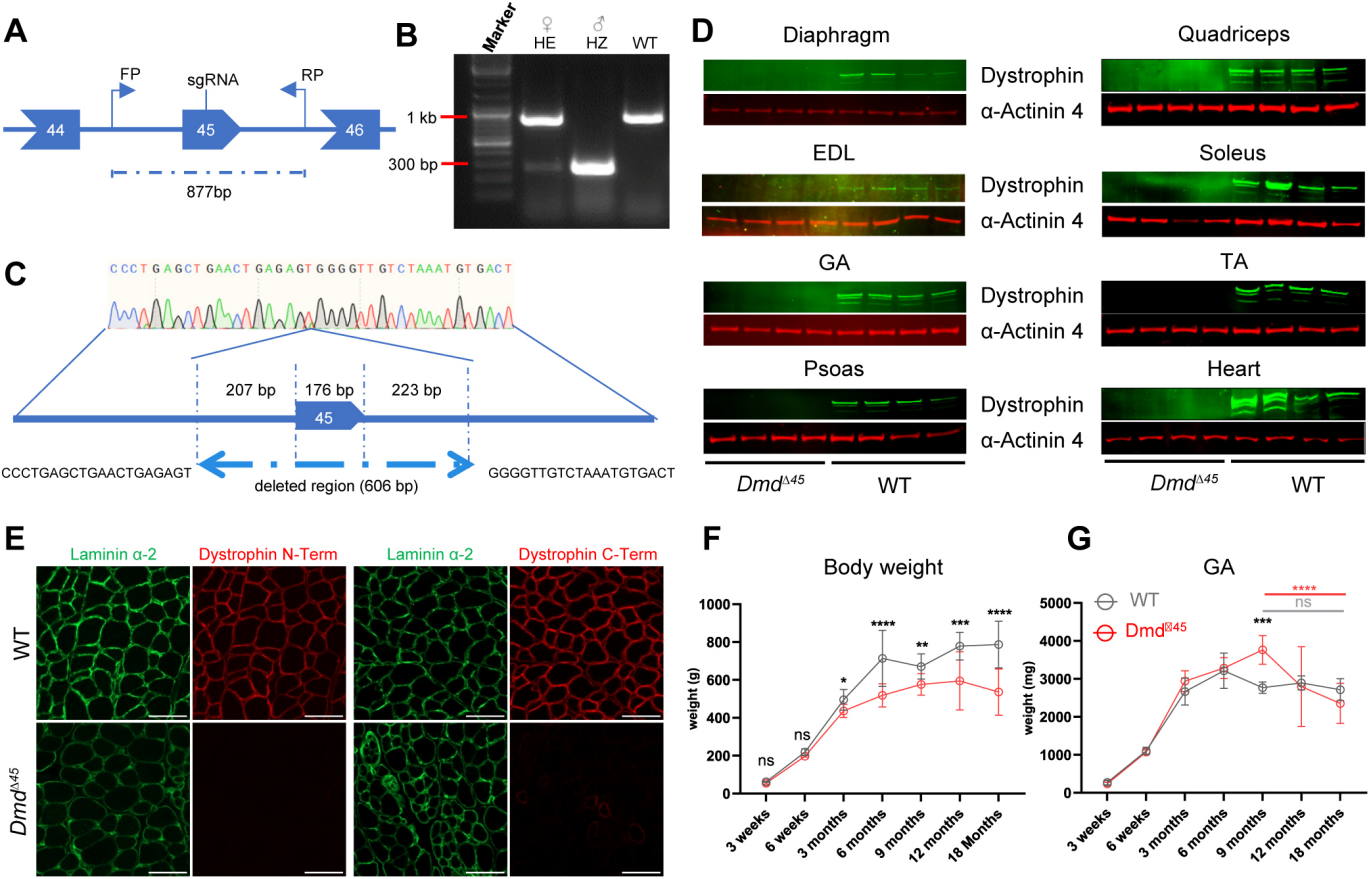
***Dmd^Δ45^* rats lack dystrophin expression, and show progressive weight loss and late muscle atrophy.** (A) Schematic representation of the targeted *Dmd* locus in *Dmd*^Δ*45*^ rats using a CRISPR-based strategy with a single guide RNA. FP, forward primer; RP, reverse primer used for genotyping. (B) Genotyping of *Dmd*^Δ*45*^ rats. ♀ HE female heterozygous; ♂ HZ, male hemizygous; WT, wild type. (C) Schematic representation of the 606-bp deletion in the *Dmd*^Δ*45*^ rat model at the targeted locus. (D) Western blot analysis for dystrophin expression in the diaphragm, extensor digitorum longus (EDL), gastrocnemius (GA), psoas, quadriceps, soleus, tibialis anterior (TA) and heart tissues (Heart) from 3-week-old *Dmd*^Δ*45*^ and WT rats (*n*=4). α-Actinin 4 was used as loading control. (E) Representative images of EDL muscle cross-sections from 3-week-old WT and *Dmd*^Δ*45*^ rats immunostained for laminin α-2 and dystrophin, using antibodies targeting the *N*- and C-terminal regions of dystrophin. Scale bars: 50 µm. (F) Body weight measurement of WT and *Dmd*^Δ*45*^ rats aged between 3 weeks and 18 months (*n*=3–16, mean±s.d.). (G) GA muscle weight measurement of WT and *Dmd*^Δ*45*^ rats aged between 3 weeks and 18 months (*n*=3-10*,* mean±s.d.). Two-way ANOVA was performed with factors genotype and age, followed by Šidák correction of multiple comparisons. Black asterisks indicate significant differences between genotypes at the same age. Significant differences across ages within the same genotype are shown in genotype-matched colors, i.e. gray for WT and red for *Dmd*^Δ*45*^. ANOVA *P*-values, ns, not significant; *P<0.05, **P<0.01, ***P<0.001, ***P=0.0001.

To generate the *Dmd*^Δ45^ rat model, we used a CRISPR/Cas9 system to delete exon 45 of the rat dystrophin gene, which has an exon organization and reading frame identical to the human gene, including the mutational hotspot-region-spanning exons 45−55 (Ensembl Reference ENSG00000198947 and ENSRNOG00000046366 for human and rat, respectively). A single-guide RNA (sgRNA) was designed to target exon 45 (Fig. 1A) and was inserted in the SpCas9/gRNA vector for injection into Sprague Dawley zygotes. This CRISPR/Cas9 approach generated a double-stranded break in exon 45, which then exploits the error-prone nature of non-homologous end-joining (NHEJ) yielding a large deletion. The selected founder line carried a deletion of 606 bp encompassing exon 45, as confirmed by genotyping and Sanger sequencing (Fig. 1B,C). We predicted potential off-target sites using CRISPOR (https://crispor.gi.ucsc.edu/crispor.py) against the rat reference genome (mRatBN7.2). We found no sites with one to three mismatches but identified 82 potential sites with four mismatches, including four located within exons, i.e. *Shroom2*, *Wfdc2*, *Misfa*, and *Dmrtc1b* (Supplemental Dataset 1). By using primers flanking these off-target exon regions (Table S1), we amplified and, subsequentially, sequenced the products using Sanger sequencing (Fig. S1A). We did not detect single nucleotide polymorphisms, insertions or deletions (indels), except for a single nucleotide polymorphism (SNP) in the first intron, which was present in both WT and *Dmd*^Δ*45*^ rats (Fig. S1B) and, thus, unrelated to off-targeting.

The loss of dystrophin expression in skeletal and cardiac muscles from 3-weeks-old *Dmd*^Δ45^ rats, detected by western blot, confirmed disruption of the *Dmd* reading frame (Fig. 1D). Immunostaining of extensor digitorum longus (EDL) skeletal muscle sections with dystrophin-specific antibodies targeting the C- and N-terminals further validated the absence of dystrophin protein expression (Fig. 1E).

The *Dmd*^Δ45^ rats exhibited moderately reduced lifespan. Among 146 wild-type (WT) and 151 *Dmd*^Δ45^ rats bred during the project, nine *Dmd*^Δ45^ rats died between 3 and 18 months of age, whereas no WT rats died before 18 months. Total body weights of *Dmd*^Δ45^ rats, recorded between 3 weeks and 18 months of age, were compared to age-matched WT controls (*n*=3–16 per group). *Dmd*^Δ45^ rats displayed reduced weight gain starting at 3 weeks, which persisted throughout their lifespan (Fig. 1F). Interestingly, weight analysis of individual skeletal muscles, including gastrocnemius (GA), EDL, tibialis anterior (TA) and soleus, showed no significant differences in weight between *Dmd*^Δ45^ rats and WT rats at early timepoints (3 weeks, 6 weeks, 3 months, 6 months) (Fig. 1G and Fig. S2). A significant increase in skeletal muscle weight was observed in *Dmd*^Δ45^ at 9 months, followed by a gradual significant decline between 9 and 18 months only in *Dmd*^Δ45^ rats. By contrast, WT rats maintained stable skeletal muscle weights over this period. These findings suggest a late-onset, progressive muscle atrophy in the *Dmd*^Δ45^ rat model.

### Histological characterization shows key dystrophic features in the skeletal muscle

**Fig. 2.**
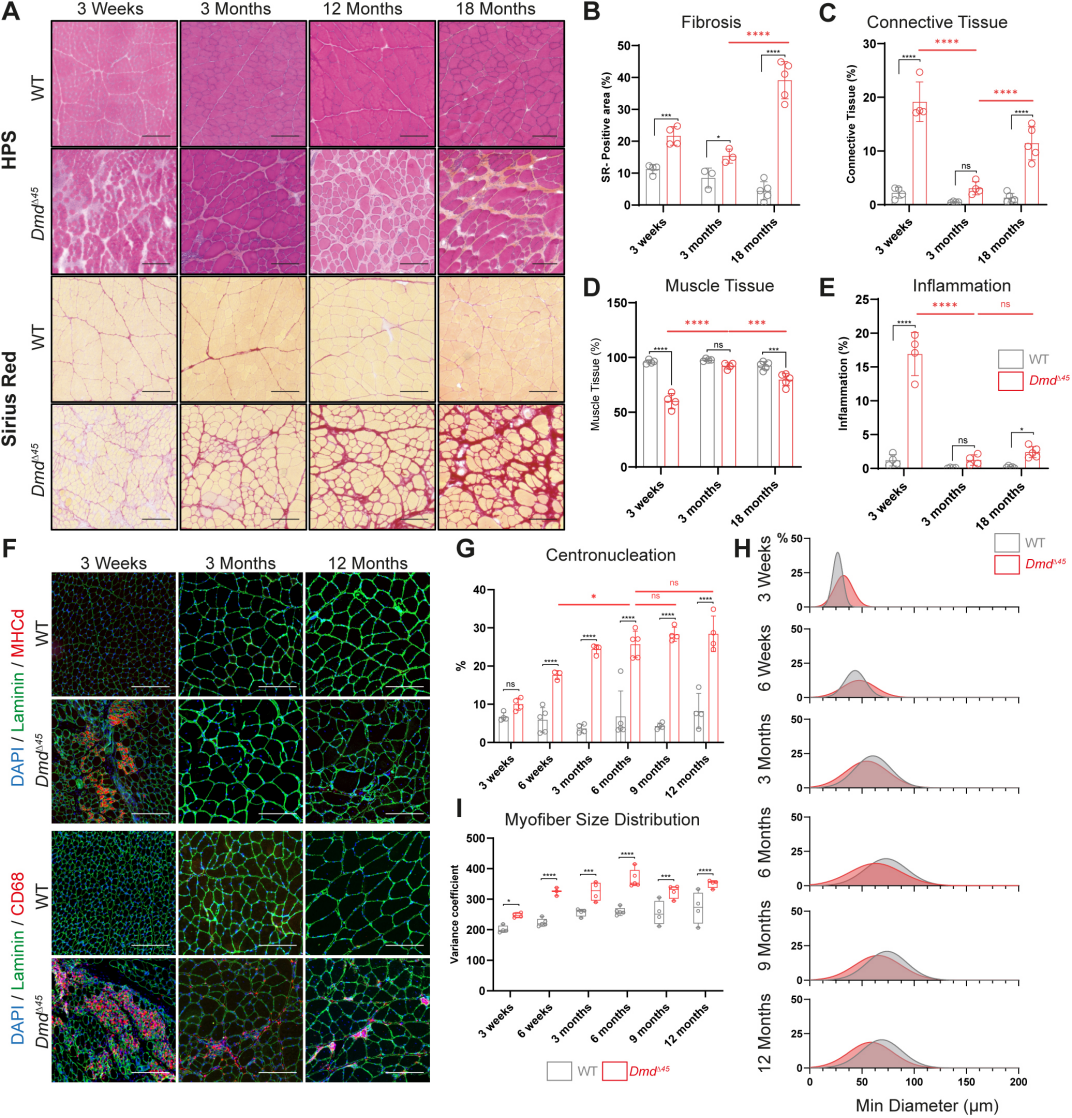
**Histological evaluation of quadriceps muscles reveals key dystrophic features in *Dmd^Δ45^* rats.** (A) Representative images of HPS and SR stainings of quadriceps cross-sections. Scale bars: 200 µm. (B) Quantification of fibrosis based on Sirius Red (SR)-positive area (mean±s.d., *n*=3–5 rats, 2–3 whole-muscle cross-sections quantified per rat). (C–E) Quantification from HPS-stained cross-sections (mean±s.d., *n*=3–5, 2–3 whole-muscle cross-sections quantified per rat) of (C) connective tissue percentage, (D) muscle tissue percentage and (E) inflammatory area percentage. (F) Representative images of quadriceps cross-sections immunostained for Laminin /MHCd or Laminin/CD68. Scale bars: 200 µm. (G) Quantification of the centronucleation (*n*=3–5 per group, with 2–3 whole-muscle cross-sections per rat, mean±s.d.). (H) Density plots depicting myofibre minimum diameter size distribution between 3 weeks and 12 months. (I) Analysis of myofibre size distribution in the GA (box plots showing median+ minimum/maximum). Coefficient of variance is calculated as: [standard deviation of the muscle fibre size/mean of muscle fibre size] *1000. Two-way ANOVA was performed with factors genotype and age, followed by Šidák correction of multiple comparisons. Significant differences between genotypes at the same age are indicated with black asterisks, while significant differences across ages within *Dmd*^Δ*45*^ rats are shown in red. ANOVA *P*-values; ns, not significant; *P<0.05, **P<0.01, ***P<0.001, ***P=0.0001.

Next, we set out to characterize the histological features of skeletal muscle at early to later stages of disease progression. Hematoxylin Phloxine Saffron (HPS) and Sirius Red (SR) staining on quadriceps (QUA) muscles showed hallmark dystrophic features as early as 3 weeks of age. (Fig. 2A). Notably, fibrosis was prominent at this early stage (Fig. 2A,B), particularly in degenerative regions, suggesting a reactive fibrotic response to muscle breakdown. This was evidenced by light pink SR staining of oedema-like regions and increased connective tissue deposition in HPS-stained sections (Fig. 2C), as well as a reduced muscle area (Fig. 2D). Interestingly, fibrosis and connective tissue content decreased temporarily at 3 months in the quadriceps, potentially due to active muscle regeneration. However, progressive interstitial fibrosis developed thereafter, culminating in extensive fibrotic remodelling at 18 months, as observed for both SR and HPS staining, and corresponding quantifications (Fig. 2A-C). This fibrotic progression was also noticed in other skeletal muscles, including the GA and EDL (Fig. S3A,B). HPS staining additionally revealed substantial inflammatory infiltrates at 3 weeks (Fig. 2A,E, Fig. S3A,B). This peak in inflammation was partially resolved over time, not only in the quadriceps but also in other skeletal muscles (Fig. 2E, Fig. S3B). Immunostaining for CD68, a marker of monocyte lineage cells, confirmed these findings, with a high abundance of CD68-positive infiltrates at 3 weeks, which were diminished at 3 months and scarce at 12 months (Fig. 2F).

To assess muscle regeneration, we performed immunostaining for developmental myosin heavy chain (MHCd) (Fig. 2F). At 3 weeks, the presence of numerous MHCd-positive myofibres suggested early activation of muscle regeneration, likely triggered by the initial wave of muscle degeneration. This regenerative response diminished over time, as indicated by a marked reduction in MHCd-positive myofibres. Concurrently, the percentage of muscle tissue increased between 3 weeks and 3 months (Fig. 2D, Fig. S3B). Centronucleation, a key marker of muscle regeneration, increased with age and stabilized ∼6 months in the quadriceps and other skeletal muscles (Fig. 2G, Fig. S3C). This aligned with the MHCd-staining patterns that showed early positivity at 3 weeks but reduced later, indicative of an initially robust regenerative phase that eventually plateaued.

Compared to WT rats, analysis of myofibre size revealed an early increase within *Dmd*^Δ*45*^ rats at 3 weeks (Fig. 2H), followed by a progressive decline, ultimately leading to significantly smaller myofibres in dystrophic muscles. This pattern correlated with the muscle weight analysis (Fig. 1G, Fig. S2), which showed an initial increase in muscle mass followed by progressive loss, suggesting late-onset muscle atrophy. Coefficient of variance analysis further highlighted increased heterogeneity in myofibre size distribution from 3 weeks onwards (Fig. 2I), a pattern consistent with disease progression.

Overall, the histological characterization of the quadriceps and other skeletal muscles in *Dmd*^Δ*45*^ rats revealed hallmark features of progressive muscular dystrophy. The disease course was characterized by an initial phase of muscle degeneration and inflammation, triggering an early regenerative response. However, this was followed by progressive muscle deterioration, marked by interstitial fibrosis and muscle atrophy.

### Elevated circulating biomarkers and declining muscle function in *Dmd*^Δ45^ rats

**Fig. 3.**
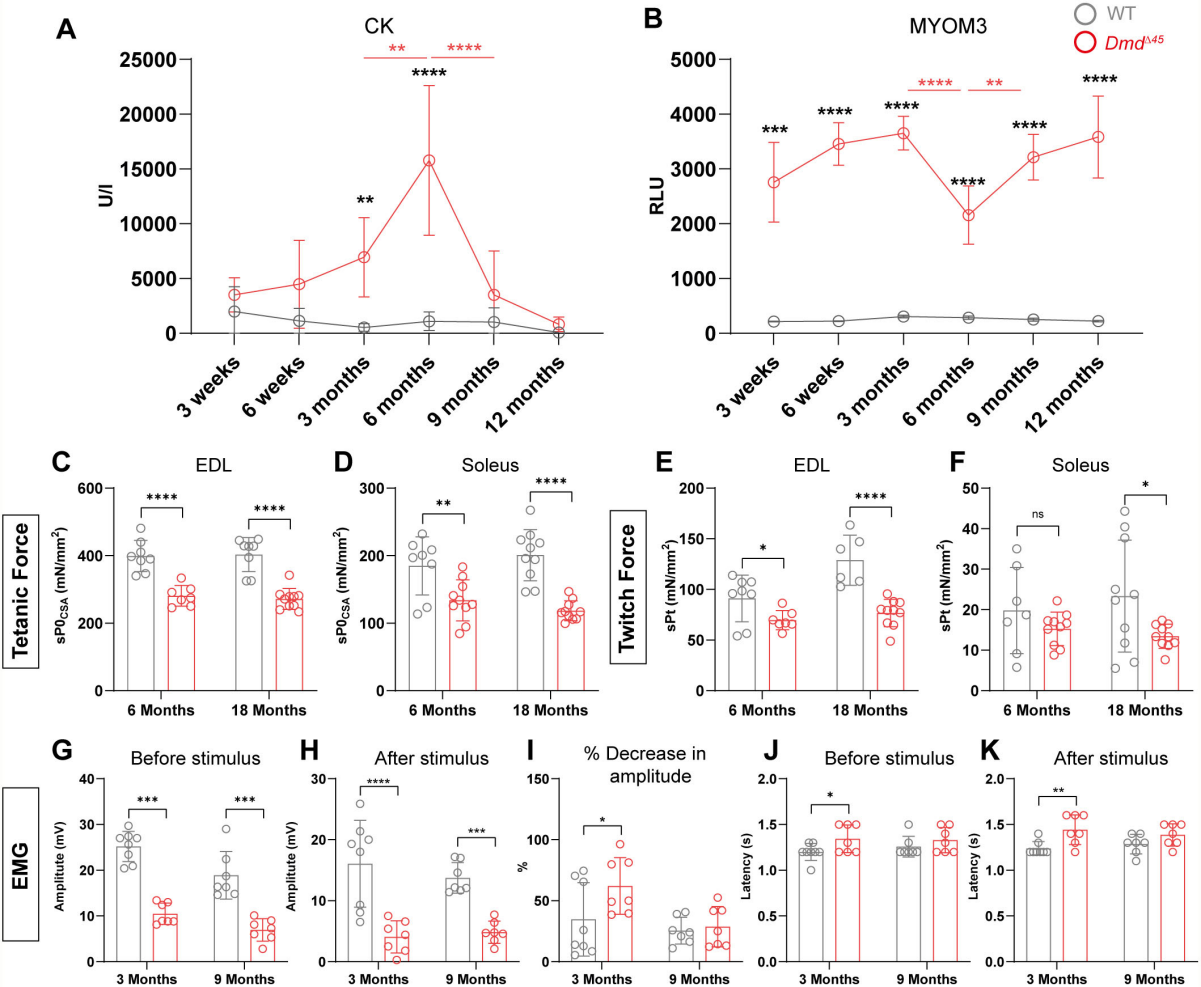
**Elevated muscle damage biomarkers and impaired muscle force indicate muscle dysfunction in *Dmd^Δ45^* rats.** (A) Measurement of serum creatine kinase (CK) activity in rats between 3 weeks and 12 months (*n*=4–5 per group, mean±s.d.). (B) ELISA quantification of myomesin-3 (MYOM3) fragments in rat serum between 3 weeks and 12 months (*n*=4–5 per group, mean±s.d.). (C–F) *In vitro* mechanical force measurements in EDL and soleus muscles from 6- and 18-month-old rats (*n*=7–10 per group, mean±s.d.). sP0csa, maximal tetanic force normalized to muscle cross-sectional area; sPt, maximal twitch force normalized to muscle cross-sectional area. (G,H) Electromyography (EMG) analysis of the gastrocnemius in 3- and 9-month-old rats, showing the amplitude of the compound muscle action potential (CMAP) before and after stimulus trains (4×200 stimuli at 10 Hz) (*n*=7–8 per group, mean±s.d.). (I) Bar graph depicting the percentage decrease in CMAP amplitude following stimulus (*n*=7-8, mean±s.d.). (J,K) Bar graphs showing latency, i.e. the time required for the electrical impulse to travel from the nerve to the muscle, before and after stimulus (*n*=7-8, mean±s.d.). Two-way ANOVA was performed with factors genotype and age, pairwise comparisons between the two time points were tested using Fisher's LSD. Significant differences between genotypes at the same age are indicated with black asterisks, while significant differences across ages within the same genotype are shown in genotype-matched colours (grey for WT, red for *Dmd*^Δ*45*^). ANOVA *P*-values, *P<0.05, **P<0.01, ***P<0.001, ***P=0.0001.

To monitor disease progression, we assessed various serum biomarkers at multiple time points. CK, a widely used marker of myofibre damage, was measured first. CK levels (U/l) were significantly elevated in *Dmd*^Δ45^ rats by 3 months and increased further at 6 months before declining between 6 and 9 months (Fig. 3A). This age-related decline in CK levels mirrors observations in patients, where levels of CK decrease as motor function deteriorates and muscle mass is replaced by fibro-fatty tissue (Bez Batti Angulski et al., 2023; Duan et al., 2021). We also measured circulating levels of myomesin 3 (MYOM3) protein fragments, a more specific biomarker of myofibre damage in muscular dystrophies compared to CK (Rouillon et al., 2015). MYOM3 levels were statistically significantly elevated in *Dmd*^Δ45^ rats compared to WT controls starting at 3 weeks, with further increase observed until 3 months (Fig. 3B). Interestingly, MYOM3 levels followed a different trajectory compared with those of CK, decreasing at 6 months, and rising again between 6 and 9 months.

To evaluate skeletal muscle function in the *Dmd*^Δ45^ rat model, we evaluated the mechanical force generation *ex vivo* in EDL and soleus muscles isolated from 6- and 18-month-old *Dmd*^Δ45^ rats. We first measured the tetanic force, which represents the maximum sustained force generated by a muscle during continuous high-frequency electrical stimulation. In the EDL, *Dmd*^Δ45^ rats exhibited a statistically significant reduction in tetanic force compared to WT controls at both ages, generating ∼70% of the average maximum tetanic force normalized to muscle cross-sectional area produced by WT muscle (Fig. 3C). In the soleus, however, the reduction was less pronounced at 6 months (134 mN mm^−2^ in *Dmd*^Δ45^ vs 185 mN mm^−2^ in WT) but became more pronounced by 18 months (124 mN mm^−2^ in *Dmd*^Δ45^ vs 201 mN mm^−2^ in WT) (Fig. 3D). We also assessed twitch force, defined as the force generated by a single, brief contraction in response to a single electrical stimulus. In EDL muscles, *Dmd*^Δ45^ rats showed statistically significantly reduced maximum twitch force at both 6 months (70 mN mm^−2^ in *Dmd*^Δ45^ vs 91 mN mm^−2^ in WT) and 18 months (77 mN mm^−2^ in *Dmd*^Δ45^ vs 129 mN mm^−2^ in WT) (Fig. 3E). In the soleus, there was no statistically significant difference in twitch force between the groups at 6 months (Fig. 3F). However, by 18 months, a significant reduction in twitch force was observed in *Dmd*^Δ45^ rats (13 mN mm^−2^ in *Dmd*^Δ45^ vs 23 mN mm^−2^ in WT). It is noteworthy that, unlike the EDL, the soleus muscle contains a majority of slow-twitch myofibres, which predominantly consists of fast-twitch fibres. This composition likely explains why the soleus muscle is less affected at 6 months, as slow-twitch fibres are known to be preserved the earlier stages of DMD (Webster et al., 1988). Overall, these findings reveal a progressive decline in both tetanic and twitch force in *Dmd*^Δ45^ rats, suggesting a gradual loss of the capacity of the muscle for sustained force production during prolonged contractions and its ability to respond to rapid, isolated activations.

Furthermore, to gain insights into neuromuscular activity, we performed electromyography (EMG) on the GA muscle to evaluate its response to sciatic nerve stimulation. The amplitude of the motor response, recorded as the compound muscle action potential (CMAP), reflects the number of activated muscle fibres. CMAP amplitude was measured both before and after stimulus trains (4×200 stimuli at 10 Hz). In *Dmd*^Δ45^ rats, the CMAP amplitude, both before and after the stimulus trains, was statistically significantly reduced compared to that in WT rats at both 3 and 9 months of age (Fig. 3G,H). At 3 months, *Dmd*^Δ45^ rats exhibited a greater decrease in CMAP amplitude following stimulus trains, indicative of increased muscle fatigue (Fig. 3I). By 9 months, the decrease in CMAP amplitude after stimulation was comparable between *Dmd*^Δ45^ and WT rats. Latency, which measures the time required for the electrical impulse to travel from the nerve to the muscle and provides insights into nerve conduction, increased significantly in *Dmd*^Δ45^ rats at 3 months (both before and after stimulus trains) (Fig. 3J,K). However, no statistically significant difference in latency was observed between genotypes at 9 months (Fig. 3J,K). These findings reveal a reduction in neuromuscular activity and responsiveness to stimulation, and evidence of neuropathy in *Dmd*^Δ45^ rats. Interestingly, the observed defects were more pronounced at 3 months compared to 9 months, suggesting a potential adaptive response in the neuromuscular system over time.

### Cardiac histological and functional assessments

**Fig. 4.**
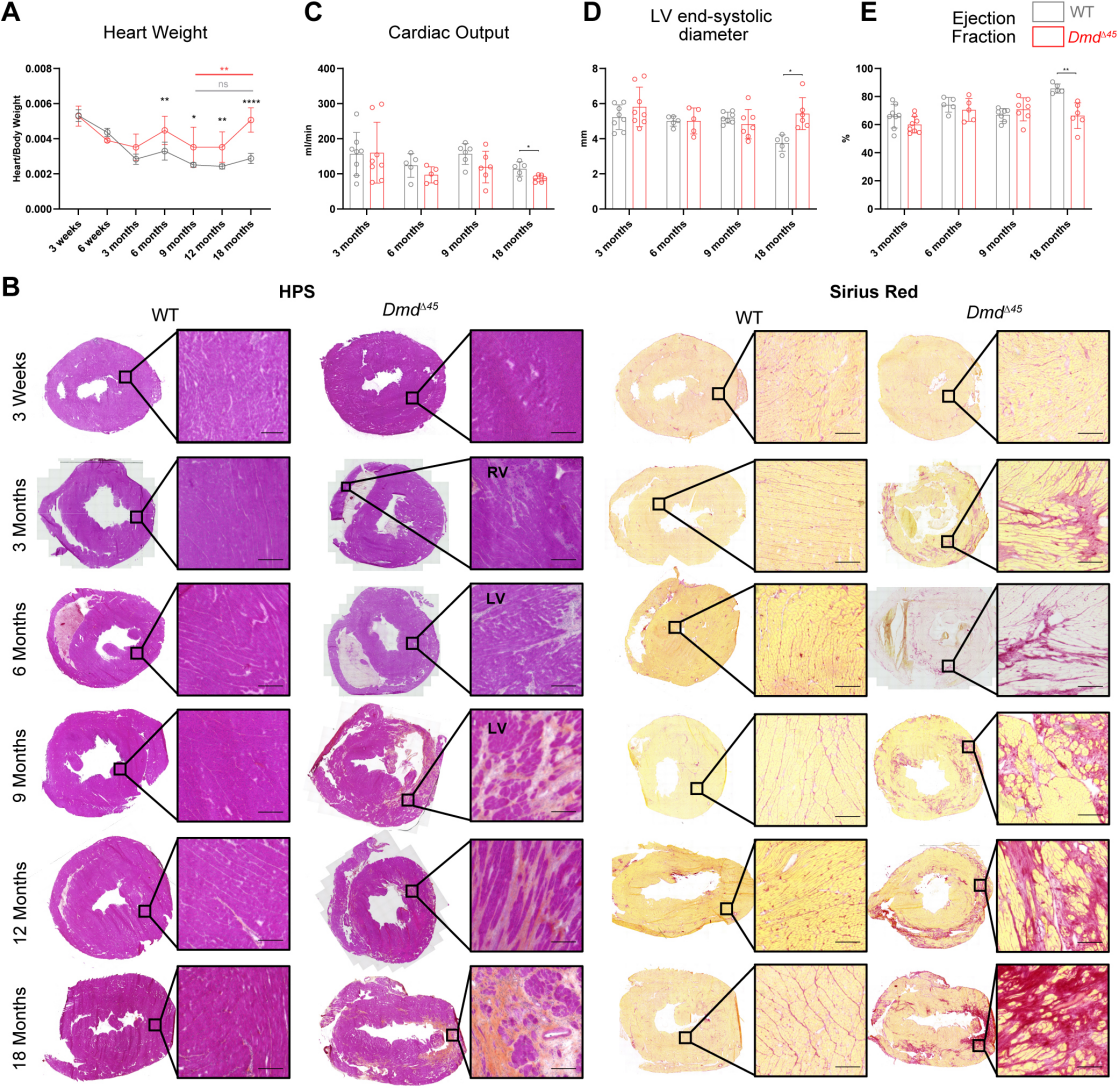
**Cardiac characterization reveals progressive cardiac pathology in *Dmd^Δ45^* rats.** (A) Heart weight normalized to body weight (*n*=3–6, mean±s.d.). (B) Histological characterization of heart tissues. The left panel shows Hematoxylin Phloxine Saffron (HPS) staining, while the right panel displays Sirius Red staining highlighting cardiac fibrosis. LV, left ventricle; RV, right ventricle. Scale bars: 200 µm. (C) Cardiac output measured by transthoracic echocardiography (TTE) in rats between 3 and 18 months of age (*n*=5–8, mean±s.d.). (D) Left ventricular (LV) end-systolic diameter measurements obtained via TTE (*n*=5–8, mean±s.d.). (E) Bar plot showing ejection fraction (*n*=5–8, mean±s.d.). Two-way ANOVA with factors genotype and age, followed by Šidák correction of multiple comparisons, was used to analyse heart weight. Unpaired two-tailed *t*-test was used for statistical comparisons of echocardiography parameters. Black asterisks indicate significant differences between genotypes at the same age. Significant differences across ages within the same genotype are shown in genotype-matched colours, i.e. grey for WT and red for *Dmd*^Δ*45*^ mice. *t*-test and ANOVA *P*-values, *P<0.05, **P<0.01, ***P<0.001, ***P=0.0001.

We next evaluated heart structure and function in *Dmd*^Δ*45*^ and WT control rats at various ages to characterize the cardiac pathology, which is currently recognized as the leading cause of death in patients diagnosed with DMD (Cheeran et al., 2017; Schultz et al., 2022). Typically, patients diagnosed with DMD develop a progressive dilated cardiomyopathy, marked by inflammatory cell infiltration, necrosis, excessive cardiac fibrosis (Olivieri et al., 2016), left ventricular dilation and progressive decline in cardiac function, ultimately leading to heart failure. To investigate this phenotype, we first monitored heart tissue weight throughout the rat lifespan. Starting at 6 months, *Dmd*^Δ*45*^ rats exhibited a statistically significant increase in heart weight normalized to body weight, compared to WT controls (Fig. 4A). Heart weight increased further significantly between 9 and 18 months only in *Dmd*^Δ*45*^ rats (1.6-fold increase compared to WT controls by 18 months).

Heart histology was assessed from 3 weeks to 18 months, using HPS and SR staining to evaluate tissue remodelling and abnormalities (Fig. 4B). At 3 weeks, no notable abnormalities were observed. However, by 3 months, inflammatory infiltrates and reactive interstitial fibrosis were evident, predominantly in the epicardial region of the right ventricle (RV). Between 6 and 9 months, aberrant structural changes emerged in the left ventricle (LV) and septum, including pronounced inflammation and dense replacement fibrosis, with the LV being more severely affected than the RV. Fibroid deposits progressively increased until 18 months, at which point a substantial portion of the heart tissue, including both the RV and LV, was composed of dense fibrotic networks.

Cardiac function and structure were further evaluated through transthoracic echocardiography (TCE) at 3, 6, 9 and 18 months, with colour Doppler imaging performed at 3 and 9 months to gain additional insights into cardiac flow patterns, velocities and pressures (Table S2). At 3 months of age, no changes in LV structure or function were detected in *Dmd*^Δ*45*^ rats. However, a statistically significant increase in pulmonary artery (PA) and pulmonary valve (PV) parameters was noticed, including increased PA velocity, pressure gradient, pressure and pulmonary ejection time compared to WT controls. These findings indicate pulmonary hypertension, predictive of right heart failure. This correlates well with histological observations showing greater pathological changes in the RV than the LV at this age (Fig. 4B). At 6 months of age, no changes in systolic function were observed, but there was a slight – albeit statistically not significant – reduction in stroke volume and cardiac output, possibly due to reduced RV preload (Fig. 4C). At 9 months of age, echocardiography and Doppler imaging revealed a decrease in several LV hemodynamic parameters, including aortic velocity, pressure gradient, pressure, stroke volume and cardiac output. These changes were indicative of LV diastolic dysfunction, characterized by prolonged LV relaxation time (LVRT) and increased LV filling pressures (Table S2). These abnormalities led to tricuspid valve insufficiency, RV failure and signs of heart failure with preserved ejection fraction (HFpEF). At 18 months of age, statistically significant decreases in ejection fraction (EF) and increased LV end-systolic diameter (LVESD) indicated systolic dysfunction with decreased contractility (Fig. 4D,E), consistent with heart failure with reduced ejection fraction (HFrEF). Increased end-systolic volume (ESV) and statistically significantly reduced cardiac output were observed (Fig. 4C). However, LV end-diastolic diameter (LVEDD) and end-diastolic volume (EDV) did not increase, indicating the absence of compensatory mechanisms, such as increased filling. Overall, the cardiac characterization of *Dmd*^Δ*45*^ rats revealed a progressive cardiac pathology that mirrors key features observed in patients diagnosed with DMD, including the transition from RV-dominant abnormalities and pulmonary hypertension in early stages to LV dysfunction and heart failure at later stages.

### Behavioural evaluation of *Dmd*^Δ45^ rats show cognitive impairments

**Fig. 5.**
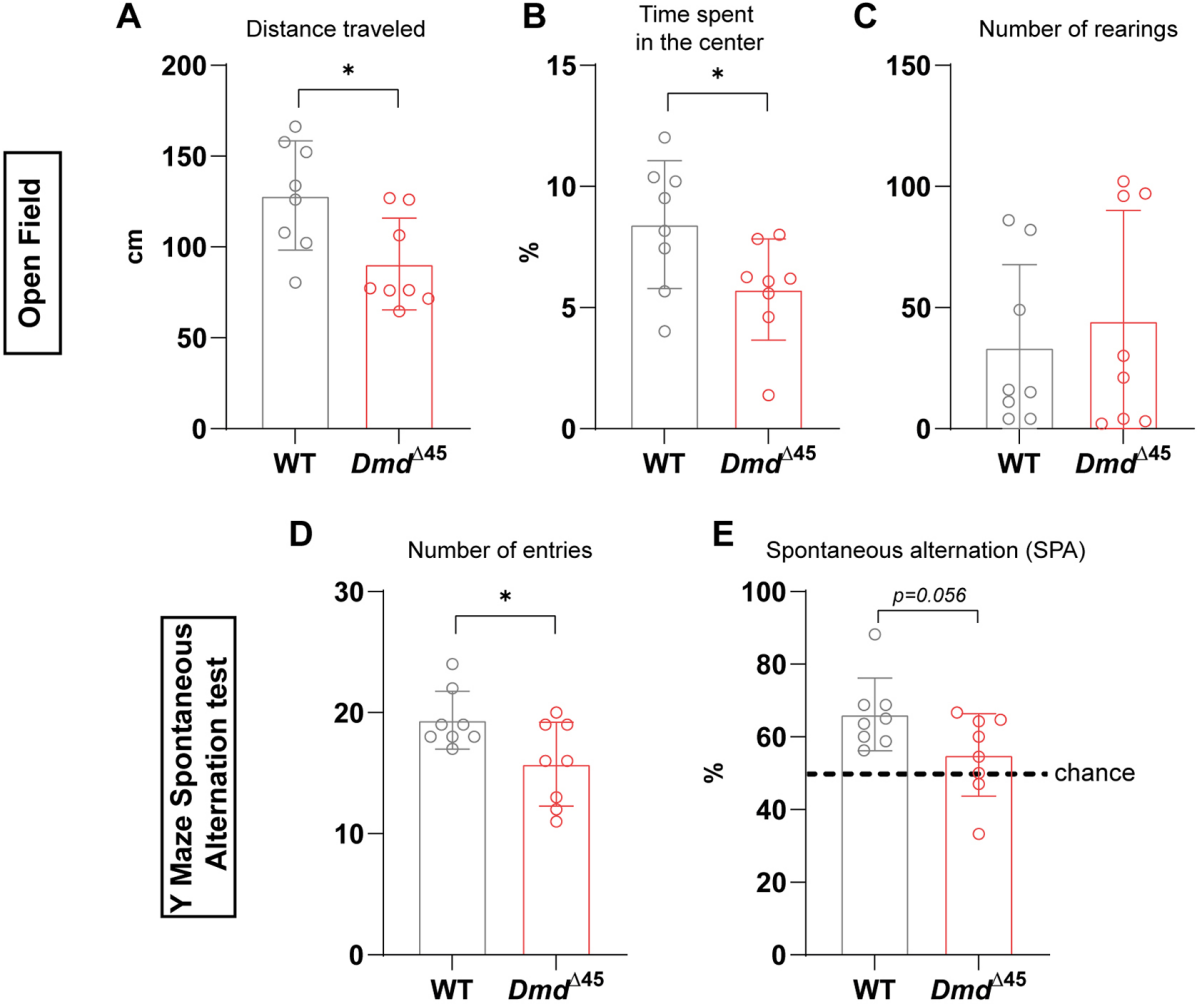
**Behavioural assessment reveals cognitive deficits in *Dmd^Δ45^* rats**. (A–C) Open field test in 3-month-old WT and *Dmd^Δ45^* rats, analysing (A) total distance travelled, (B) percentage of time spent in the centre, and (C) number of rearings (*n*=8 per group, mean±s.d.). (D,E) Y-maze spontaneous alternation test, quantifying the total number of arm entries (D) and the spontaneous alternation (SPA) percentage between arms (E) (*n*=8, mean±s.d.). Unpaired two-tailed *t*-test was used for statistical comparisons (*P<0.05).

Cognitive impairments, including non-progressive deficits with diverse manifestations, have been reported in many patients diagnosed with DMD (Anderson et al., 2002; Banihani et al., 2015; Rae and O'Malley, 2016). To evaluate behavioural and cognitive function as well as locomotor activity in *Dmd*^Δ45^ rats, we conducted a series of tests on 3-month-old rats. The open field test, a widely used assay for assessing exploratory behaviour, general locomotor activity and anxiety-related responses, revealed noticeable differences between *Dmd*^Δ45^ rats and WT controls. *Dmd*^Δ45^ rats exhibited a statistically significantly decreased total distance travelled (Fig. 5A), indicating reduced exploratory behaviour and locomotor activity. Additionally, they spent less time in the centre of the arena and more time along the periphery (Fig. 5B), suggesting increased anxiety or decreased curiosity. However, no differences were observed in the number of rearing events (Fig. 5C), indicating that this aspect of exploratory behaviour remained intact.

To assess spatial and working memory, we employed the Y-maze spontaneous alternation test, in which rats were allowed to explore a Y-shaped maze freely for 8 min. *Dmd*^Δ45^ rats showed a reduced number of arm entries, consistent with decreased locomotor activity and signs of anxiety (Fig. 5D). Furthermore, their spontaneous alternation percentage, a measure of working memory based on the proportion of consecutive entries into all three arms without repetition, was lower than WT controls (Fig. 5E). Although this decrease did not reach statistical significance (*P*=0.056), the alternation percentage was significantly higher than chance only (50%) in WT rats, indicating impaired working memory. Overall, these findings suggest that *Dmd*^Δ45^ rats exhibit cognitive and behavioural impairments, including reduced exploratory behaviour, heightened anxiety-related responses and deficits in working memory, which align with previously reported symptoms in other DMD rat models (Larcher et al., 2014).

### RNA sequencing analysis indicates dysregulated pathways related to *DMD*

To gain insight into the consequences of exon 45 deletion in *Dmd*^Δ45^ rats at molecular level, we performed RNA sequencing (RNA-seq) on skeletal (psoas, soleus and diaphragm) and cardiac muscles from both 6- and 12-month-old rats (complete data are provided in the Supplemental Dataset 2). First, we quantified the reads of each exon of the *Dmd* gene, confirming the deletion of exon 45 (Fig. S4A) and the dramatic decrease of the expression of the *Dmd* gene, in all muscle samples (Fig. S4B). We further analysed exon usage in the *Dmd* gene by grouping exons into two regions: ‘Beginning’ (upstream of exon 45) and ‘End’ (downstream of exon 45). We found a progressive reduction in *Dmd* transcript levels decreasing from Beginning to End exons (Fig. S4C) in accordance with the previously described transcriptional imbalance in patients diagnosed with DMD (Spitali et al., 2013). Given that utrophin (*Utrn*) can partially compensate for the loss of dystrophin, we assessed its expression and observed significant upregulation over time in skeletal muscles, with a modest, statistically not significant increase in the heart (Fig. S4D).

**Fig. 6.**
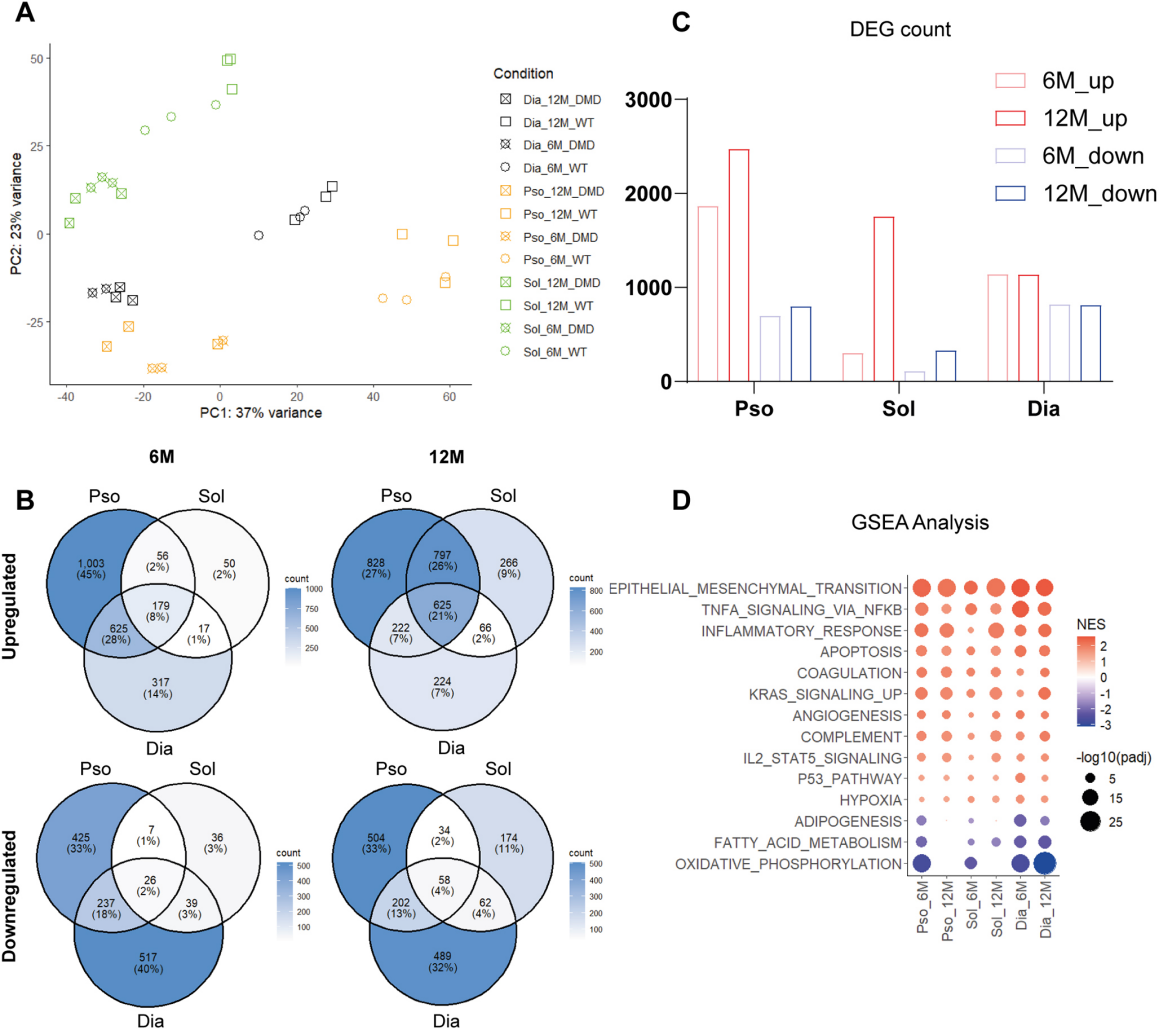
**Transcriptomic analysis of dysregulated pathways in *Dmd*^Δ*45*^ rats.** (A) Principal component analysis (PCA) of gene expression profiles. PC1, predominantly genotype-specific variation; PC2, genotype and tissue-specific variation. (B) Venn diagrams showing the overlap of differentially expressed genes (DEGs), including up- and downregulated genes, across skeletal muscles (Pso, Sol, Dia) in *Dmd*^Δ*45*^ rats compared with WT at 6 and 12 months. Pso, psoas; Sol, soleus; Dia, diaphragm. Colour intensity corresponds to the number of genes in each category. (C) Bar plot illustrating the total number of the DEGs in each muscle. (D) Dot plot of commonly enriched pathways. Each dot represents a pathway in a specific *Dmd*^Δ*45*^ muscle; dot size reflects -log10(adjusted *P*-value) and dot colour indicates the Normalized Enrichment Score (NES). Pathways are ordered by the average NES across all muscles.

We then performed principal component analysis (PCA) on skeletal muscle tissues to reduce data complexity. PCA revealed a clear separation between *Dmd*^Δ45^ and WT groups, with PC1 capturing mostly genotype-driven differences and PC2 reflecting both genotype and tissue-specific variation (Fig. 6A). Notably, the transcriptomic divergence between *Dmd*^Δ45^ and WT samples exceeded the variation among different skeletal muscle types. To identify genes contributing most to these differences, we averaged the PC1 contribution scores (i.e. the weights, indicating how strongly each gene influences PC1) across all skeletal muscle RNA-seq datasets at both timepoints. Genes with an average contribution score >0.03 were selected, representing those most strongly associated with genotype-driven transcriptional changes across muscles and timepoints. This approach identified 36 key dysregulated genes (33 upregulated and three downregulated) (Fig. S5 and Table S3). Half of these had been previously reported as dysregulated in DMD, such as *MymX*, *Mymk*, *Spp1*, *Thbs4*, *Postn*, *Myl4*, *Timp1* and *Aqp4* (Patsalos et al., 2024), while the others were novel in this context (e.g. *Igfn1*, *Draxin*, *Lrrc15*, *TMEM184a*, *Ptx4* and *Cilp* for the upregulated genes, and *Barx2* and *Mylk4* for the downregulated ones).

Volcano plots highlighting globally dysregulated genes (fold-change >2, adjusted *P*-value <0.05) are shown in Fig. S6A. Among skeletal muscles, the diaphragm exhibited the most-pronounced dysregulation (with the fold chance exceeding 1000), followed by the psoas and soleus, in agreement with histological findings. Venn diagrams summarize the overlap of differentially expressed genes (DEGs) across muscles at both time points (Fig. 6B). At 6 months, 179 genes were upregulated across three muscles, with 56 additional genes shared by psoas and soleus. By 12 months, shared upregulated genes rose to 625, with 797 more shared between psoas and soleus. As for downregulated genes, 25 were common across all muscles at 6 months (excluding *Dmd*), with seven genes shared by psoas and soleus. By 12 months, this number increased to 58 common downregulated genes, and 34 shared between psoas and soleus. A temporal analysis of DEG evolution (Fig. 6C, Tables S4,S5, Fig. S6A) suggested an overall age-dependent worsening, with the exception of the diaphragm. A total of 127 and 11 genes were found to be commonly upregulated and downregulated, respectively, across all skeletal muscles at both 6 and 12 months (Table S4). Among the commonly downregulated genes, *Barx2* was also identified within the list of PC1-associated genes. Similarly, 20 of the commonly upregulated genes overlapped with the PC1 gene list. In total, 21 out of the 36 PC1-associated genes overlapped with the set of commonly dysregulated genes, underscoring the effectiveness of our strategy in identifying DMD-relevant genes. Among the commonly dysregulated genes that were not PC1-associated, several have previously been reported in the context of DMD, including *Dlk1* (Zhang et al., 2019) *Fgf23* (Wehling-Henricks et al., 2016), *Fgf7* (Fernandez-Simon et al., 2024), *Thbs2* (Elasbali et al., 2024), *Adam12* (Jorgensen et al., 2007) and *Sfrp1* (Sohn et al., 2015). By contrast, other dysregulated genes, such as *Ccn4*, *Ltbp2*, *Tmem119*, *Sema3a*, *Tp73*, *Mepe*, *Col19a1*, *Col8a1*, *Col16a1*, *Cthrc1*, *Fibin*, *Erbb2*, *Steap2*, *Cfh* and *Nfkbiz* had not previously been linked to DMD and may represent novel candidates for further investigations.

Next, we performed Gene Set Enrichment Analysis (GSEA) on skeletal muscle RNA-seq by using Hallmark gene sets from the MSigDB database (adjusted *P*<0.05) (Fig. 6D, Fig. S6B). Overall, the most-significantly dysregulated hallmark pathways were upregulated and included epithelial to mesenchymal transition, inflammation and immune responses, and stress- or death-associated processes (e.g. apoptosis, DNA repair). By contrast, downregulated pathways primarily involved metabolic processes, such as adipogenesis, fatty acid metabolism and oxidative phosphorylation. At 12 months, the upregulated pathways remained largely consistent compared to those at 6 months, while downregulated pathways became more concentrated on fatty acid metabolism.

**Fig. 7.**
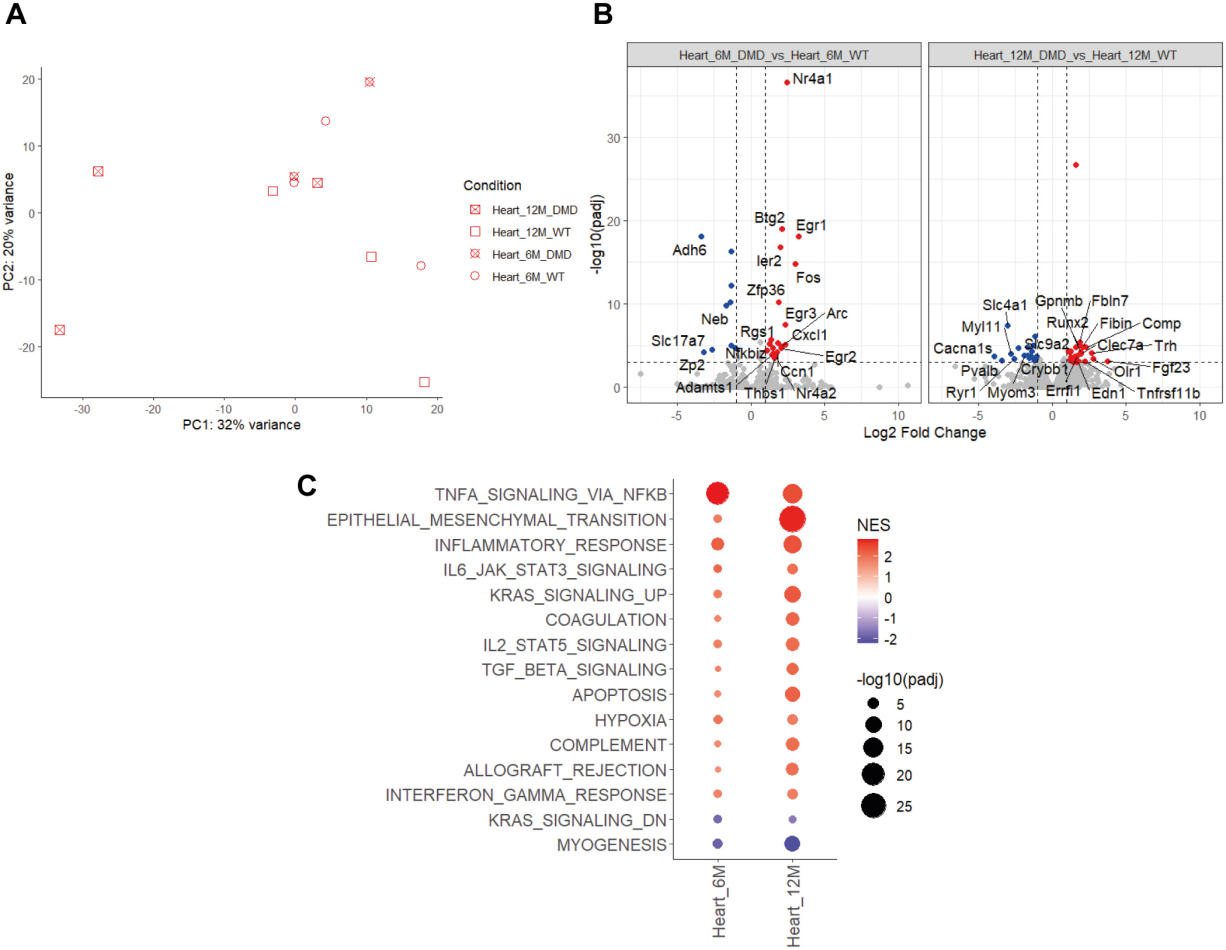
**Transcriptomic analysis of dysregulated pathways in the heart of *Dmd*^Δ*45*^ rats.** (A) PCA scatter plot of gene expression profiles. (B) Volcano plots showing DEGs in *Dmd*^Δ*45*^ at 6 and 12 months. (C) GSEA highlighting differentially enriched Hallmark pathways at 6 months and 12 months.

Regarding the heart, PCA analysis revealed no clear separation between WT and *Dmd*^Δ45^ rats at 6 months. However, at 12 months, the transcriptomic profiles of two out of three *Dmd*^Δ45^ rats were distinct from WT controls, potentially reflecting inter-individual variability in the onset or progression of cardiomyopathy (Fig. 7A). Differential expression analysis identified 35 and 231 upregulated genes, and 24 and 76 downregulated genes in *Dmd*^Δ45^ hearts at 6 and 12 months, respectively, compared to age-matched WT controls (Fig. 7B, Table S6). The DEGs were enriched in pathways related to inflammation and immune responses (e.g. *Cxcl1*, *Socs3*, *Nfkbiz*, *Selplg*), fibrosis and ECM remodelling (*Thbs1*, *Adamts1*, *Serpine1*, *Ccn1*) and transcriptional regulation (*Nr4a1/2*, *Bhlhe40*). By contrast, several downregulated genes were involved in muscle structure and function (*Neb*), and metabolism (*Adh6*, *Pxmp4*).

Interestingly, three genes upregulated in the heart at 12 months, *Comp*, *Gpnmb* and *Fbln7*, were also part of the PC1 gene set previously identified in skeletal muscle. *Comp* is a reported biomarker of fibrosis in a DMD rat model (Taglietti et al., 2022), while *Gpnmb* is both a marker and effector of growth factor-expressing macrophages and contributes to muscle regeneration in DMD (Patsalos et al., 2024). By contrast, *Fbln7* – which encodes fibulin-7, a member of the ECM-associated fibulin family (Chakraborty et al., 2020) – has not been previously associated with DMD. Notably, *Fbln7* was also upregulated in all skeletal muscles at both 6 and 12 months, suggesting its potential as a novel biomarker of ECM remodelling in DMD.

GSEA analysis further revealed dysregulated pathways in the heart (Fig. 7C). At 6 months, there was significant activation of immune-related pathways, with TNFα signalling and inflammatory response among the most enriched. By contrast, pathways associated with myogenesis were downregulated. By 12 months, immune activation persisted but was less pronounced compared to that at 6 months, while pathways related to ECM remodelling became more prominent. These included epithelial-mesenchymal transition, apoptosis and TGF-β signalling. Notably, the pathways altered in the heart at 12 months closely resembled those observed in the psoas muscle at 6 months, suggesting that cardiac disease progression lags behind skeletal muscle pathology.

### Spontaneous exon skipping partially restores dystrophin expression in *Dmd*^Δ*45*^ rat model

**Fig. 8.**
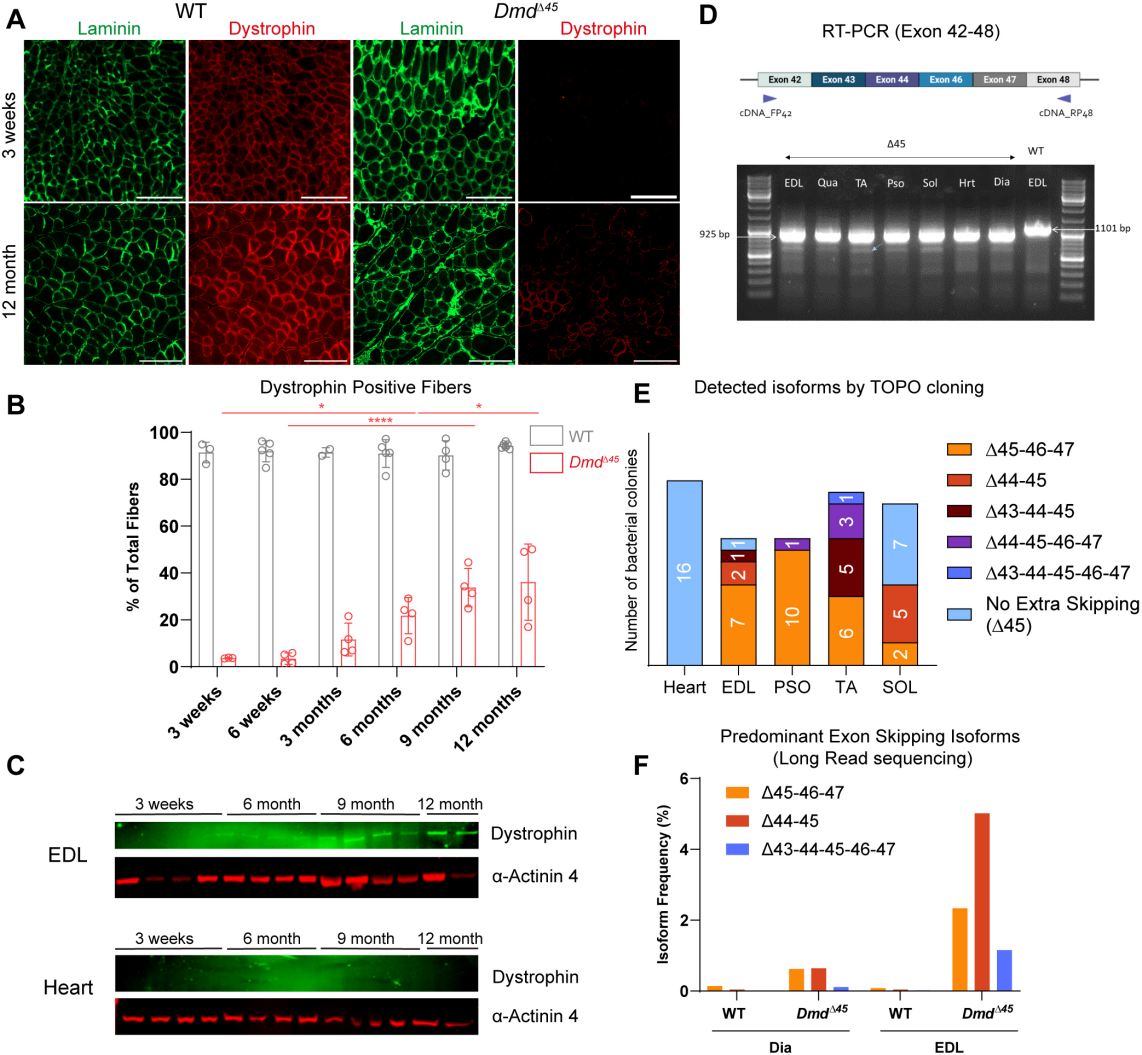
**Spontaneous exon skipping partially restore dystrophin expression in *Dmd*^Δ*45*^ rats**. (A) Immunofluorescence staining for dystrophin in EDL muscle at 3 weeks and 12 months. Scale bars: 100 µm (upper panel) and 200 µm (lower panel). (B) Quantification of dystrophin-positive myofibres of EDL muscles from 3 weeks to 12 months of age (*n*=3–5, mean±s.d.). (C) Western blot analysis of dystrophin in EDL and heart. (D) RT-PCR analysis of dystrophin exons 42–48 in muscle samples, visualized by gel electrophoresis. Hrt, heart. (E) Representation of cDNA sequences obtained via topoisomerase based (TOPO) cloning from dystrophin amplicons (exons 42–48) across different muscles. A total of 16 sequences from heart, 11 from EDL and psoas, 15 from TA, and 14 from soleus were analysed. (F) Proportion of exon-skipping events between exons 42–48 in diaphragm and EDL muscles, measured by PACBIO long-read sequencing. Two-way ANOVA was performed with factors genotype and age, followed by Šidák correction of multiple comparisons, for analysis of dystrophin positive fibres. Significant differences across ages within *Dmd^Δ45^* rats are shown in red. ANOVA *P*-values, *P<0.05, ****P<0.0001.

Although *Dmd*^Δ45^ rats exhibit skeletal muscle dystrophy and cardiomyopathy, molecular, histological and functional analyses suggested a milder phenotype compared to other published rat models, such as the ‘exon 23 deleted’ rat (Larcher et al., 2014) or the *Dmd*^Δ52^ rat model (Taglietti et al., 2022). Since all these models involve out-of-frame mutations in the *Dmd* gene, we sought to understand the basis for the phenotypic differences observed. Notably, we detected rare sporadic dystrophin-positive fibres in the EDL and other muscles from 3-week-old *Dmd*^Δ45^ rats. This prompted an investigation of revertant fibres across ages, from 3 weeks to 12 months. Quantification of revertant fibres revealed an age-dependent increase in dystrophin-positive fibres in skeletal muscles (Fig. 8A,B, Fig. S7A,B). In the EDL, the percentage of revertant fibres reached on average 36% (±16%) by 12 months of age (Fig. 8B). In TA and soleus muscles, revertant fibres were also observed, although to a lesser extent, reaching on average 29.3% (±25.5%) and 10.9% (±7.9%), respectively, at 12 months. Western blot analysis on EDL further confirmed the re-emergence of dystrophin expression, with visible dystrophin levels at 9 and 12 months, but this was not observed in the heart of *Dmd*^Δ45^ rats (Fig. 8C).

Spontaneous exon skipping, which underlines the formation of revertant fibres, has been reported in patients diagnosed with deletion of exon 45 in the *DMD* gene (Dwianingsih et al., 2014; Thanh et al., 1995; Prior et al., 1997). To investigate this mechanism in our model, we amplified dystrophin cDNA spanning exons 42–48 from skeletal muscles and hearts of 6-month-old WT and *Dmd*^Δ45^ rats. In WT muscle, a single 1101-bp band corresponding to the full-length sequence was observed (Fig. 8D). By contrast, in *Dmd*^Δ45^ muscles, in addition to the expected 925 bp band lacking exon 45, we detected additional band of lower molecular weight (∼700 bp and less). The main and smaller bands were isolated and purified, then subjected to topoisomerase based (TOPO) cloning to separate different amplicons into bacterial colonies. Subsequent Sanger sequencing of selected colonies confirmed the presence of multiple isoforms (each colony corresponding to one isoform) (Fig. 8E). In the heart, all 16 colonies analysed corresponded to the same isoform (deletion of exon 45). In skeletal muscles, however, five distinct isoforms were identified, with the predominant sequence in EDL, psoas and TA corresponding to the deletion of exons 46–47. Overall, these results showed that multiple exons were skipped in the skeletal muscle but not in the heart. Importantly, in three out of five detected isoforms, exon skipping restored the *Dmd* reading frame (Fig. S7C), explaining the expression of truncated dystrophins and presence of revertant fibres in skeletal muscles but not the heart. To confirm and quantify these events without colony-selection bias, we performed PacBio® long-read sequencing of amplicons from exons 42–48 obtained from WT and *Dmd*^Δ45^ EDL and diaphragm muscles. In EDL, the most frequent isoforms (other than the exon 45 deletion) involved skipping of exon 44 (5.02% of transcripts), skipping of exons 46–47 (2.34%) and a larger multi-exon skip spanning exons 43–47 (1.16%) (Fig. 8F). In diaphragm, the top three isoforms were similar, although detected at lower frequencies. Altogether, these findings demonstrate that the *Dmd*^Δ45^ model recapitulates spontaneous exon-skipping events similar to those reported in patients with exon 45 deletions.

To explore the molecular mechanism underlying exon skipping in skeletal muscles of *Dmd*^Δ45^ rats, we analysed the expression of spliceosome-related genes from our RNA-seq dataset by using the MSigDB gene set. We identified 49 spliceosome-related genes that were significantly dysregulated (adjusted *P*-value <0.050), the majority of which being downregulated in the skeletal muscles of *Dmd*^Δ*45*^ rats (Fig. S6D). By contrast, none of these genes were differentially expressed in the heart at 6 or 12 months, a result consistent with the absence of revertant fibres and exon skipping events in cardiac tissue. This suggests that a general downregulation of spliceosome components can lead to impaired spliceosome assembly, facilitating exon skipping selectively in skeletal muscle of *Dmd*^Δ*45*^ rats.

## DISCUSSION

In this study, we generated a *Dmd*^Δ45^ rat model by deleting exon 45 of the *Dmd* gene, which represents the most frequent single-exon deletion within the mutational hotspot region in patients diagnosed with DMD. This deletion results in an out-of-frame mutation, leading to the absence of dystrophin expression in both skeletal and cardiac muscle tissues by 3 weeks of age.

Phenotypically, *Dmd*^Δ45^ rats exhibited an early increase in skeletal muscle mass, which declined progressively between 9 and 18 months of age. This pattern is consistent with an initial phase of pseudohypertrophy, supported by the early accumulation of connective tissue and fibrosis, followed by late-onset progressive muscle atrophy. Histopathological analysis confirmed classical features of muscular dystrophy: early cycles of muscle degeneration and inflammation accompanied by a strong regenerative response, ultimately giving way to progressive muscle deterioration characterized by interstitial fibrosis and pronounced muscle atrophy.

This structural deterioration was also reflected functionally. *Dmd*^Δ45^ rats demonstrated a progressive decline in both tetanic and twitch force, indicating a reduction in the capacity of muscle for sustained force production as well as its responsiveness to brief activations. In parallel, we observed a decrease in neuromuscular activity and responsiveness to stimulation, along with evidence of neuropathy. Interestingly, these neuromuscular deficits were more pronounced at 3 months than at 9 months, suggesting a potential adaptive or compensatory response within the neuromuscular system over time. A limitation, however, is that EMG recordings were performed under ketamine/xylazine anaesthesia, which is known to influence neuromuscular transmission. Thus, results should be interpreted with caution, as anaesthetic effects may have contributed to the observed differences, potentially in an age-dependent manner.

Circulating biomarkers of muscle damage, including CK and MYOM3, were elevated in *Dmd*^Δ*45*^ rats compared to WT controls. Notably, these markers followed distinct temporal trajectories. CK levels peaked around 6 months and then gradually declined, whereas MYOM3 levels decreased at 6 months before increasing again thereafter. This divergence may reflect their underlying biological roles: CK, a cytosolic enzyme, is released upon sarcolemmal damage and mirrors early acute injury, while MYOM3, a structural protein, may better indicate muscle fibre necrosis and chronic damage progression.

Cardiac assessment revealed a progressive cardiomyopathy that recapitulates key clinical features of DMD, including a shift from early RV dysfunction and pulmonary hypertension to later-stage LV failure, accompanied by progressive fibrosis and cardiac remodelling. Similar manifestations have been reported in other DMD rat models (Sugihara et al., 2020; Szabo et al., 2021; Taglietti et al., 2022) and are likely to contribute to the increased mortality observed in *Dmd*^Δ*45*^ rats compared with WT controls. A limitation of our study, however, is the absence of a dedicated survival analysis with systematic necropsy to determine cause of death. In addition, longitudinal electrocardiographic monitoring would be particularly valuable, as arrhythmias and other conduction abnormalities are frequent in DMD and may represent a major driver of sudden death in rats.

Beyond muscular and cardiac phenotypes, *Dmd*^Δ*45*^ rats also exhibited cognitive and behavioural deficits, including reduced exploratory behaviour, increased anxiety-like responses and impairments in working memory. These neuro-behavioural alterations parallel the cognitive symptoms observed in patients diagnosed with DMD. Overall, this model recapitulates main aspects of the clinical symptoms observed in patients diagnosed with DMD, confirming suitability of the rat as a preclinical model, mirroring the human disease better than existing mouse models.

Compared to previously reported DMD rat models, *Dmd*^Δ*45*^ exhibited phenotypes that were similar to or more severe than those observed *Dmd*^Δ3-16^ and *Dmd*^mdx^ rats, but less severe than in *Dmd*^Δ52^ rats, which are characterized by rapid disease progression and early mortality (Taglietti et al., 2022). Specifically, the reduction in weight gain occurred earlier in the *Dmd*^Δ*45*^ rats than in *Dmd*^Δ3-16^ and *Dmd*^mdx^ rats but at a time point similar to that in *Dmd*^Δ52^ rats (Nakamura et al., 2014; Larcher et al., 2014; Taglietti et al., 2022). In terms of circulating biomarkers, CK protein levels in *Dmd*^Δ*45*^ rats were between those observed in *Dmd*^Δ3-16^/*Dmd*^mdx^ and *Dmd*^Δ52^ rats (Nakamura et al., 2014; Larcher et al., 2014; Taglietti et al., 2022), further supporting the notion of an intermediate disease severity. Cardiac abnormalities in *Dmd*^Δ52^ rats were comparable in magnitude to those reported in *Dmd^mdx^* and *Dmd*^Δ3-16^ rats (Sugihara et al., 2020), suggesting that the *Dmd*^Δ*45*^ model retains the ability to develop the full spectrum of DMD-associated cardiac pathology, which is often absent in mouse models.

The intermediate phenotype presented by *Dmd*^Δ*45*^ rats may be attributed to the presence of a relatively high number of revertant fibres, reaching on average 36% in the EDL by 12 months of age. This contrasts with *Dmd*^Δ3-16^ rats, in which revertant fibres are rare (Sugihara et al., 2020). Long amplicon sequencing revealed that spontaneous exon skipping events lead to re-framing of the *Dmd* transcript and expression of a truncated dystrophin protein. The most-prevalent transcript variant in *Dmd*^Δ45^ rats involved skipping of exon 44, followed by skipping of exons 46 and 47. These exon-skipping patterns mirror those observed in some patients diagnosed with DMD/BMD and may contribute to variability in clinical severity. For instance, in a previous study, 13 of the 133 patients with an exon 45 deletion presented with a milder BMD-like phenotype (Dwianingsih et al., 2014). In several of these cases, exon 44 skipping was identified as the mechanism allowing the partial restoration of the reading frame and dystrophin expression (Dwianingsih et al., 2014). By contrast, another patient with an exon 45 deletion who had minimal exon 44 skipping (∼1% of truncated *DMD* mRNA), showed no signs of phenotype mitigation (Prior et al., 1997). Such inter-individual variability, ranging from severe to mild phenotypes, is well documented in cases of exon 45 deletion (Tuffery-Giraud et al., 2009; Aartsma-Rus et al., 2006; Cunniff et al., 2009; Takeshima et al., 2010) and is now replicated at molecular level in our rat model.

Transcriptomic analysis of skeletal muscles in *Dmd*^Δ*45*^ rats revealed both well-established and novel pathophysiological signatures. At 6 months, upregulation of genes related to regeneration and inflammation, such as S*ohlh2*, *Igfn1*, *Cdkn2a*, *Mymk* and *Mymx*, suggests active myogenic repair and satellite cell engagement. However, elevated *Cdkn2a* expression also indicates potential cell cycle arrest, which may reflect satellite cell exhaustion or senescence. Genes involved in ECM remodelling and immune signalling, such as *Lrrc15*, *Tmem184a*, *Ptx4* and *Clec2dl1*, were also upregulated, in line with ongoing fibrosis and chronic inflammation. These pathological responses were amplified at 12 months, with increased expression of these and other genes, indicating disease progression and intensification of compensatory pathways. By contrast, several genes were consistently downregulated at both 6 and 12 months, including *Barx2*, *Asb4*, *Ppp1r1a*, *RhoU* and *Cacng7*, implicating disruption in myogenic signalling, ion transport and muscle structural maintenance. Additional downregulated genes at 12 months, such as *Unc5a*, *Slc6a2* and *Nppc*, point to worsening transcriptional repression as degeneration advances.

Overall, the transcriptional profile of skeletal muscle transitions from early stress response and regeneration at 6 months to chronic inflammation, fibrosis, and metabolic dysfunction by 12 months. Alongside established DMD markers (*Spp1*, *Thbs4*, *Postn*), novel gene candidates emerged, such as *Igfn1*, *Draxin*, *Cilp*, *Ctxn3* and *Fbln7* (upregulated), and *Barx2* (downregulated), offering new insights into disease mechanisms, biomarker discovery and therapeutic targets.

In the heart, the transcriptomic profile revealed a temporal transition from early inflammatory and stress responses at 6 months to fibrotic remodelling and metabolic dysfunction at 12 months. At the earlier stage, immediate early genes (IEGs) such as *Egr1*, *Fos*, *Nr4a1/2* and *Arc* were upregulated, indicating rapid transcriptional responses to cellular and metabolic stress. Concurrently, inflammatory mediators (*Cxcl1*, *Socs3* and *Nfkbiz*) and ECM remodelling genes (*Thbs1*, *Ccn1*, *Adamts1*) were also elevated, marking the onset of immune activation and tissue restructuring, which is consistent with a pre-fibrotic or early remodelling phase of cardiomyopathy in DMD. By 12 months, there was a widespread upregulation of fibrosis-related genes (e.g. *Comp*, *Fbln7*, *Fn1*, *Runx2*, *Abca1*), alongside the downregulation of sarcomeric, ion channel and metabolic genes (*Ryr1*, *Cacna1s*, *Pgam2)*, indicating contractile dysfunction, energy failure and advanced cardiac degeneration. Persistent upregulation of metabolic regulators (*Fgf23*, *Abca1*, *Bcat1*) suggests ongoing metabolic stress in the degenerating cardiac tissue. This transcriptomic trajectory aligns with the natural course of DMD-associated cardiomyopathy, in which an early inflammatory phase evolves into irreversible fibrotic remodelling and functional defects.

In summary, we successfully generated a *Dmd*^Δ45^ rat model that replicates the multisystemic features of DMD, including progressive skeletal muscle atrophy, cardiomyopathy and neurobehavioral impairments. The presence of a substantial proportion of revertant fibres likely contributes to the intermediate phenotype, differentiating it from more-severe models like *Dmd*^Δ*52*^. The model offers a valuable platform for studying both the molecular basis of phenotypic variability and the mechanisms of spontaneous exon skipping. Our findings highlight exon 44 skipping as the predominant re-framing event in this model, suggesting that therapeutic strategies targeting exon 44 are particularly effective in cases of exon 45 deletion. Further investigation into the mechanisms regulating spontaneous exon skipping in *Dmd*^Δ*45*^ rats could aid the development of personalized exon-skipping therapies, with direct clinical relevance for a significant subset of patients diagnosed with DMD.

## MATERIALS AND METHODS

### *Dmd*^Δ45^ rat model generation and animal care

The *Dmd*^Δ45^ model was generated in collaboration with Genoway (https://www.genoway.com/) by using a single-guide RNA (sgRNA)-Cas9 strategy. An sgRNA [5′-UAGGAAGCUUGAGUCUGCGG-3′] with a TGG protospacer adjacent motif (PAM) sequence was designed to target exon 45 of the rat *Dmd* gene. The model was generated on a Sprague-Dawley [CD^®^(SD), Crl:CD(SD)] background, with founders obtained from Charles River Laboratories (https://www.criver.com/fr). A total of 186 embryos injected with the sgRNA/Cas9 construct were re-implanted, yielding 57 viable pups. Genomic DNA from tail biopsies was screened using a two-step strategy established by Genoway: (1) PCR amplification of the targeted *Dmd* locus followed by T7 endonuclease I assay and sequencing to characterize indels; (2) Insert-specific PCR using a single-stranded oligodeoxynucleotides (ssODN)-based primer to detect potential knock-in events. Eight pups carried variants at the *Dmd* locus. Of these, four harboured alleles predicted to disrupt *Dmd* function: (i) one male founder with a 606-bp deletion encompassing *Dmd* exon 45 (Fig. 1C) and spanning from 207 bp into the 3′ region of intron 44 to 223 bp into the 5′ region of intron 45, including exon 45; (ii) one female founder with a 213-bp deletion spanning intron 44 and exon 45 (147 bp within exon 45), including the splice acceptor site, resulting in a knockout allele; (iii) two additional founders carried indels within exon 45 that caused frameshifts and premature stop codons (a male with one 5 bp substitution replacing 10 bp; (iv) another female founder with a 148 bp deletion). The remaining four mutants carried in-frame or otherwise non-disruptive indels and were not propagated. In total, four founder animals (heterozygous/hemizygous knockout lines) were bred upon sexual maturity to generate heterozygous/hemizygous *Dmd* knock-out lines. The founder line carrying the 606-bp deletion was selected for further propagation and characterization. The *Dmd*^Δ45^ line was subsequently backcrossed at least three times with WT Sprague-Dawley rats received from Charles River Laboratories. Animals were housed in an SPF barrier facility with 12-h light/12-h dark cycles, and provided with food and water *ad libitum*. All procedures were performed in accordance with European guidelines for the care and use of laboratory animals. Experimental protocols were approved by the institute's ethics committee (C2EA-51) and the French Ministry of Research (APAFIS #25388). Only male hemizygous rats were used for the phenotypic characterization of the model.

### Genotyping

Genotyping of the rats was performed using the Phire Tissue Direct PCR Master Mix (Life Technologies). Tail fragments extracted from rats were manipulated according to the supplier's protocol. Samples were subjected to PCR with selected primers (Table S7). The apparent unequal amplification of the two bands in heterozygous females is most likely due to X-inactivation in females, as previously reported (Warnecke et al., 1997, 2002), where the inactive X is methylated and can result in reduced PCR amplification.

### Histology

Muscle and heart tissues were frozen in isopentane and stored at −80°C. Transverse cryosections (8−10 µm) were prepared from frozen tissues and stored at −80°C until staining. Histological staining with Hematoxylin Phloxine Saffron (HPS) and Sirius Red (SR) was performed according to standard procedures. All bright-field labelled histological images were digitized using an Axioscan Z1 slide scanner (Zeiss, Germany) with a Zeiss Plan-Apochromat 10×/0.45 M27 dry lens (Zeiss, Germany). Tiled scanner images were reconstructed using ZEN software (Zeiss, Germany) (pixel size=0.45 µm).

QuPath (version 0.4.3) software was used for to quantify muscle fibrosis after SR staining. For each muscle scan, a small artificial neural network was trained to classify positive and negative fibrotic pixels and, subsequently, to quantify fibrotic areas. HPS images were used to train a small artificial neural network to classify positive and negative pixels for three categories: connective tissue, muscle tissue and inflammation.

### Immunohistochemistry, immunofluorescence and image analysis

Tissue sections were dried for 5 min and rehydrated in PBS followed by blocking in either 10% goat serum or 10% foetal bovine serum (FBS) with 5% goat serum for 30 min. Primary and secondary antibodies (Table S8) were diluted in 1% goat serum (Agilent) or 1% FBS with 0.5% goat serum. The slides were then washed in PBS solution (three times for 5 min), rinsed in Milli-Q H_2_O and mounted with DAPI Fluoromount-G mounting medium (Southern Biotech). Once the slides had dried, they were scanned using the Axioscan Z1 slide scanner (Zeiss, Germany) under a Zeiss Plan-Apochromat 10×/0.45 M27 dry lens (Zeiss, Germany) with an ORCA-Flash4.0 CMOS digital camera (Hamamatsu, Japan). Tile scanner images were reconstructed using ZEN software (Zeiss, Germany) (pixel size=0.65 μm).

Fluorescence intensity, shape and size were measured for each object (fibres, fibre membrane, nuclei) using QuPath (version 0.3.2). Centronucleation was estimated by measuring the distance between each nucleus and its closest membrane coordinate, and normalized to the minimal Feret diameter of the fibre. Fibres with at least one internal nucleus located >1/5 of the minimal Feret diameter away from the membrane were classified as centronucleated. Positive fibres for each channel were detected based on the fluorescence distribution within negative control sections obtained under known negative conditions. Dystrophin-positive fibres were determined by thresholding based the relative intensity ratio between membrane and cytoplasmic staining, ensuring that 90% of fibres were classified as positive within WT muscles. Two to three whole-muscle cross-sections were analysed and averaged per rat (*n* in quantifications refers to biological replicates).

### Protein isolation and western blotting

Harvested frozen muscle and heart tissues submerged in isopentane were lysed with RIPA buffer (Thermo Fisher Scientific) containing EDTA-free protease inhibitor cocktail (Roche; 1 tablet per 10 ml RIPA buffer) and Benzonase (Sigma-Aldrich) diluted 1:1000. Primary and secondary antibodies are listed in Table S8. Membranes were revelated with the Odyssey CLx scanner (LI-COR). Images were analysed using Image Studio Lite (v. 5.2), pixels of selected bands were quantified, and the signal was normalized against reference protein.

### RNA extraction and RT-PCR

Total RNA was extracted from frozen muscle samples or sections. Samples were lysed in NucleoZOL solution (Macherey-Nagel) using the BeadMill 24 (Fisherbrand) (6 m/s – 40 s). DNAse.RNAse-free H_2_O was added to the lysed samples and total RNA was extracted using the Nucleomag extraction kit (Machery Nagel) according to the supplier's protocol. Extracted RNA samples were treated for DNA removal using the TURBO DNA-free™ kit and ribonuclease inhibitor RNasin (Promega) according to the supplier's protocol. Reverse transcription (RT) PCR of mRNA was performed with the RevertAid H Minus First Strand cDNA synthesis kit (Thermo Fisher Scientific) following the provider's protocol. The obtained cDNA was then diluted 1:4 using DNase/RNase-free H_2_O and amplified with a reverse transcription PCR reaction (RT-PCR) using Phusion^®^ High-Fidelity DNA Polymerase (New England Biolabs) with different melting temperatures corresponding to the different primers used.

### RNA-seq analysis

Total extracted RNA was quantified using a Nanodrop spectrophotometer (ND8000 Labtech, Wilmington, DE) and RNA quality (RIN≥7) was controlled using an Agilent RNA 6000 Pico Kit on a 2100 Bioanalyzer (Agilent Technologies). RNA from three aliquots was sequenced with a sequencing depth between 18 M and 50 M reads per sample. The sequencing libraries were prepared using the TruSeq Stranded Total RNA Library Prep Kit (Illumina) and sequenced according to the Illumina protocol. Paired-end reads (2×150 bp) were aligned on the reference transcriptome (Rattus_norvegicus. mRatBN7.2) using STAR version 2.7.11b (Dobin et al., 2013) with an average alignment of 95.0%, after excluding samples showing at the same time a low number of reads and a low percentage of alignment. Gene expression was measured by featureCounts version 2.0.6 (subread package). Quantification files were processed using R (4.4.1) to perform differential expression analysis. Pairwise comparisons of group differential expression were performed using DESEqn (1.44.0), considering genes with >50 reads per samples, i.e. lfcThreshold=0, pAdjustMethod=FDR, independentFiltering=T (lfc, log_2_ fold change; padj, adjusted *P*-value; FDR, false discovery rate; T, true). Read counts were normalized using the median-of-ratios method implemented in the DESeq2 package (R). Genes were considered dysregulated when the absolute log_2_-fold change was >1 and the FDR-adjusted *P*-value <0.05.

### Long-read sequencing

Purified cDNA corresponding to exons 42 to 48 obtained from wild-type (WT) or *Dmd*^Δ45^ muscle samples was isolated from gel-purified cDNA amplicons. The amplicons were ligated to SMRTbell adapters according to the manufacturer's instructions (Pacific Biosciences, Menlo Park, CA). Libraries were individually prepared for each sample and sequenced using the PacBio^®^ Sequel II platform, employing single-molecule real-time (SMRT) sequencing technology. Circular consensus sequencing (CCS) reads were generated to enhance base accuracy. Raw sequence data were processed using the PacBio SMRT Link software (version 6.0) for demultiplexing, filtering, and quality control.

### Serum biomarker quantification

Quantification of CK protein serum levels in was performed using a FUJI DRI-Chem nx500 analyser (DMV Imaging). Diluted serum (10 μl) was deposited on FUJI DRI-CHEM CPK-PIII plates (DMV Imaging), and CK protein concentrations were automatically generated in international units per litre of serum (U/l).

Enzyme-linked immunosorbent assay (ELISA) was performed on serum to quantify MYOM3 by using a Meso Scale Discovery assay (MSD) according to a previously described protocol (Palmieri et al., 2024). Briefly, the multi-array plate containing electrodes (MSD) was first coated overnight at 4°C with the capturing antibody against MYOM3 (17692-1-AP, Proteintech). After washes with PBS supplemented with 0.05% Tween 20, saturation of the plate was achieved by adding 3% bovine serum albumin (BSA) 1 h at RT. New washes were performed followed by the incubation of the diluted sera (in 1% BSA solution, 2 h at RT with agitation). To calculate the concentration of MYOM3, a standard range of a MYOM3 peptide corresponding to the antibody epitope (synthesized by ProteoGenix, Schiltigheim, France) was used. Used for detection was an in-house anti MYOM3 antibody (Clone 51-H1-B4, subcontracted to ProteoGenix) coupled with sulfo/TAG (Meso Scale Diagnostics, Rockville, MA).

### Measurement of isolated muscle force

Animals were euthanized by intraperitoneal injection of a lethal dose (0.35 ml/kg) of Dolethal (Centravet, Maisons-Alfort, France). EDL and soleus muscles were then dissected and placed in an oxygenated Tyrode buffer bath (Sigma Aldrich, 9.8 g/l, pH ∼7.4; Aurora Scientific System) at room temperature to maintain physiological conditions. Each muscle was mounted between bipolar electrodes and attached to a force transducer. Tetanic contraction was elicited by delivering 600 mA stimulations (500 ms/125 Hz for EDL and >2.4 s/90 Hz for soleus). For twitch force measurements, a single 600 mA pulse (equivalent to one action potential) was applied. Maximal forces recorded were normalized to the cross-sectional area of the muscle, calculated using the optimal muscle length Lo.

### Electromyography

Electromyographic recording allows neurophysiological measurement of motor and sensory function. Rats were anaesthetized by intraperitoneal injection (injection volume=2 ml/kg; ketamine concentration =100 mg/kg; xylazine concentration=10 mg/kg). Electromyographic recording was performed with an EMG device (NATUS, Merignac, France). Needle electrodes (30G) were used to stimulate the nerve and receive the electrical response at the muscle. Needles were inserted 1-2 mm into the skin of the animal. Four repetitive stimulation trains, each consisting of 200 electrical pulses (0.1 ms, 8 mA) at a frequency of 10 Hz, were applied to the sciatic nerve with an interval of 1 s before and after the stimulations. The amplitude of the CMAP at the level of the GA muscle was measured 30 s before and 30 s after the stimulations with a single stimulus (8 mA, 0.1 ms). The percentage decrease in the amplitude of the muscle response between the response before and after the stimulations is used as a measure of the level of muscle fatigue.

### Echocardiography

TCE images were captured using the VisualSonics Vevo 2100 (or Vevo 3100LT) Imaging System (Toronto, Canada) with a MX201 probe (18-MHz, image depth: 39 mm; image axial resolution: 100 μm; focal depth: 18 mm) or MX550D probe (15-MHz). Allowed by the echographer were M-mode, B-mode and Doppler imaging with ECG, and respiratory gating of all images. Hair from the thorax was removed by shaving and hypoallergenic cream hair remover (NairTM) to optimize the acoustic interface. Rats were anesthetized with 1–2% isoflurane in oxygen at supine position on the heating pad of the Vevo system to maintain normothermia while continuously monitoring of heart rate and rectal temperature. Prewarmed ultrasound gel was applied on the thorax. The VisualSonics rail system was used to fix the probe, avoiding any compression of the thorax. Cardiac morphology and ventricular systolic function were acquired by M-mode tracings of the left ventricle (LV) by using the short axis view, with the ultrasound beam perpendicular to the LV at the midpapillary level to determine the ejection fraction (EF), fractional shortening, wall thickness, LV inner diameter (LVID) and LV mass, with the LVM calculated as LVM =1.055×[(EDD+ SW +PW)^3^−EDD^3^], where EDD is the LV end-diastolic diameter, SW is the interventricular septal wall thickness, and PW is the posterior wall thickness. Aortic dimensions were determined using B-mode imaging in the parasternal long axis views. Alternatively, the long parasternal axis view at the level of the largest LV diameter (i.e. at the level of the papillary level) was also used to determine LV dimensions.

### Open field test

Rats were tested in automated 90×90×40 cm open fields (Panlab, Barcelona, Spain), each virtually divided into central and peripheral regions. The open field arena was placed in a room homogeneously illuminated at 15 lx. Each rat was placed within the periphery of the open field and allowed to explore the apparatus freely for 30 min, with the experimenter out of the animal's sight. Distance travelled, number of rearings and time spent in the central and peripheral regions were recorded over the test session (peripheral width=20 cm). Number of entries and percentage of time spent in centre area were used as index of emotionality/anxiety.

### Y-maze spontaneous alternation test

Testing occurs in a Y-shaped maze with three white opaque plastic arms (90×25×40 cm) at a 120° angle from each other. Each arm has specific patterns on the walls. After introduction to the centre of the maze, the animal was allowed to freely explore the three arms for 8 min. Over the course of multiple arm entries, the animal should show a tendency to enter a less recently visited arm. Arm entries and the number of triads were counted and recorded to calculate the percentage of alternation. Placement of all four limbs within a maze arm was defined as one entry.

### Statistics

Statistical analysis was performed using either GraphPad Prism 10 (GraphPad Software, Inc., La Jolla, CA) or RStudio (v4.4.1). Equality of variances was assumed, and group normality was assessed using the Shapiro–Wilk test. For longitudinal data across ages, two-way ANOVA was used with genotype and age as factors. Post-hoc analyses included (i) comparisons between genotypes within each age group and (ii) comparisons between ages within each genotype. Šidák correction was applied when more than two time points were compared; Fisher's least significant difference (LSD), equivalent to an uncorrected pairwise test, was used when only two time points were present. For independent comparisons across time points (i.e. not repeated), unpaired Student's *t*-test was used to compare means between two groups. The statistical tests applied are indicated in each figure legend. Results were considered statistically significant at *P*<0.05.

## Supplementary Material

10.1242/dmm.052578_sup1 Supplementary information

Dataset 1. List of predicted off-target sites for the sgRNA.

Dataset 2. List of DEGs from RNA sequencing in skeletal and cardiac muscles at 6 and 12 months.
