# Supplementary material for: A new dystrophin-deficient rat model mirroring exon skipping in patients with DMD exon 45 deletions
Source: Dis Model Mech. 2026 Feb 4;19(1):dmm052578. doi: 10.1242/dmm.052578 (PMC12919952; doi:10.1242/dmm.052578)
Supplement: Supplementary information [file dmm-19-052578-s1.pdf]

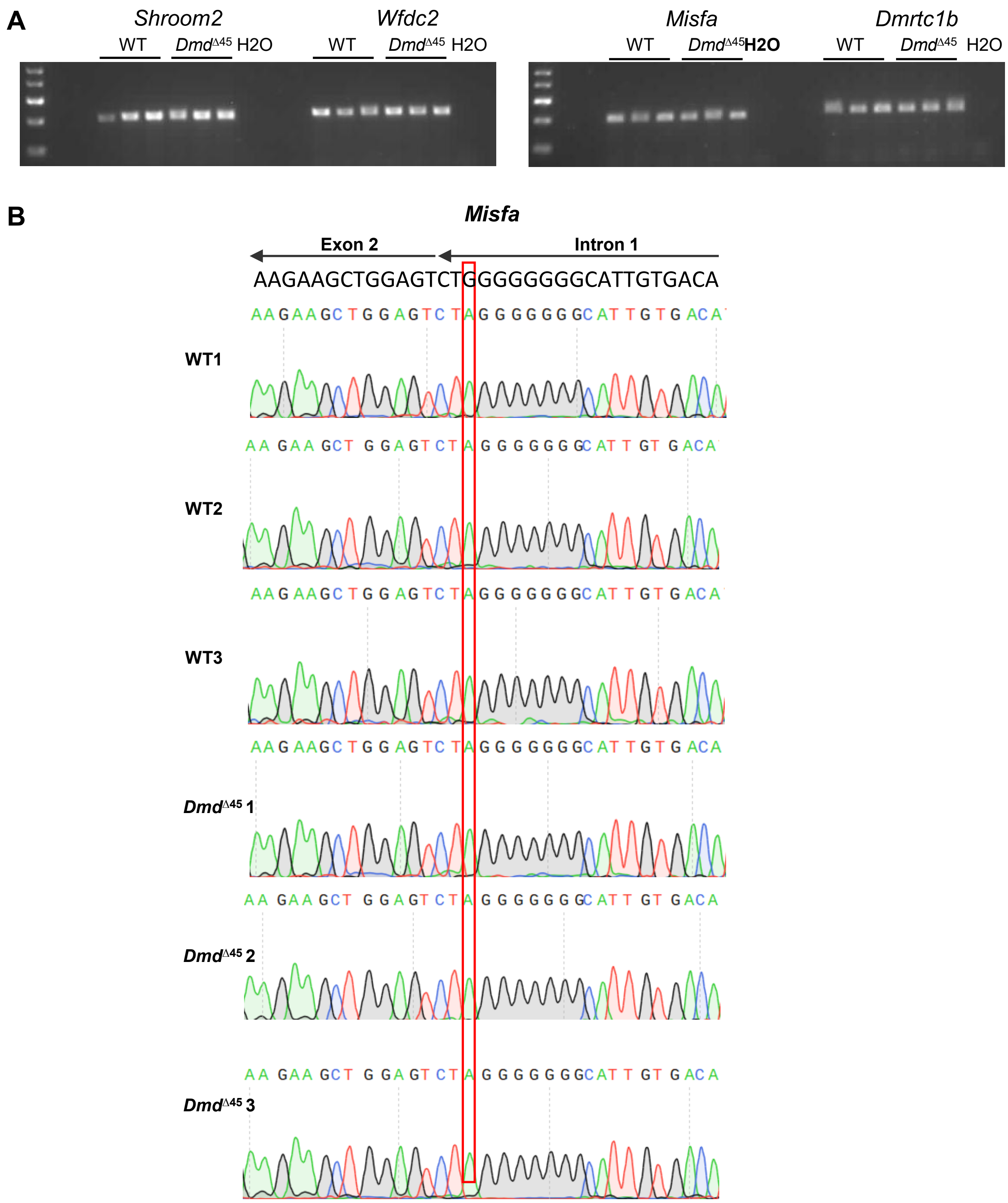

Fig. S1. Off-target site analysis in *Dmd*$^{\Delta 45}$ rats. (**A**) Representative agarose gel images of PCR amplifications from predicted off-target exonic sites**.** (**B**) Sanger sequencing identified a single nucleotide polymorphism in *Misfa* present in both WT and *Dmd*$^{\Delta 45}$ rats, confirming it is unrelated to CRISPR editing.

Disease Models & Mechanisms • Supplementary information

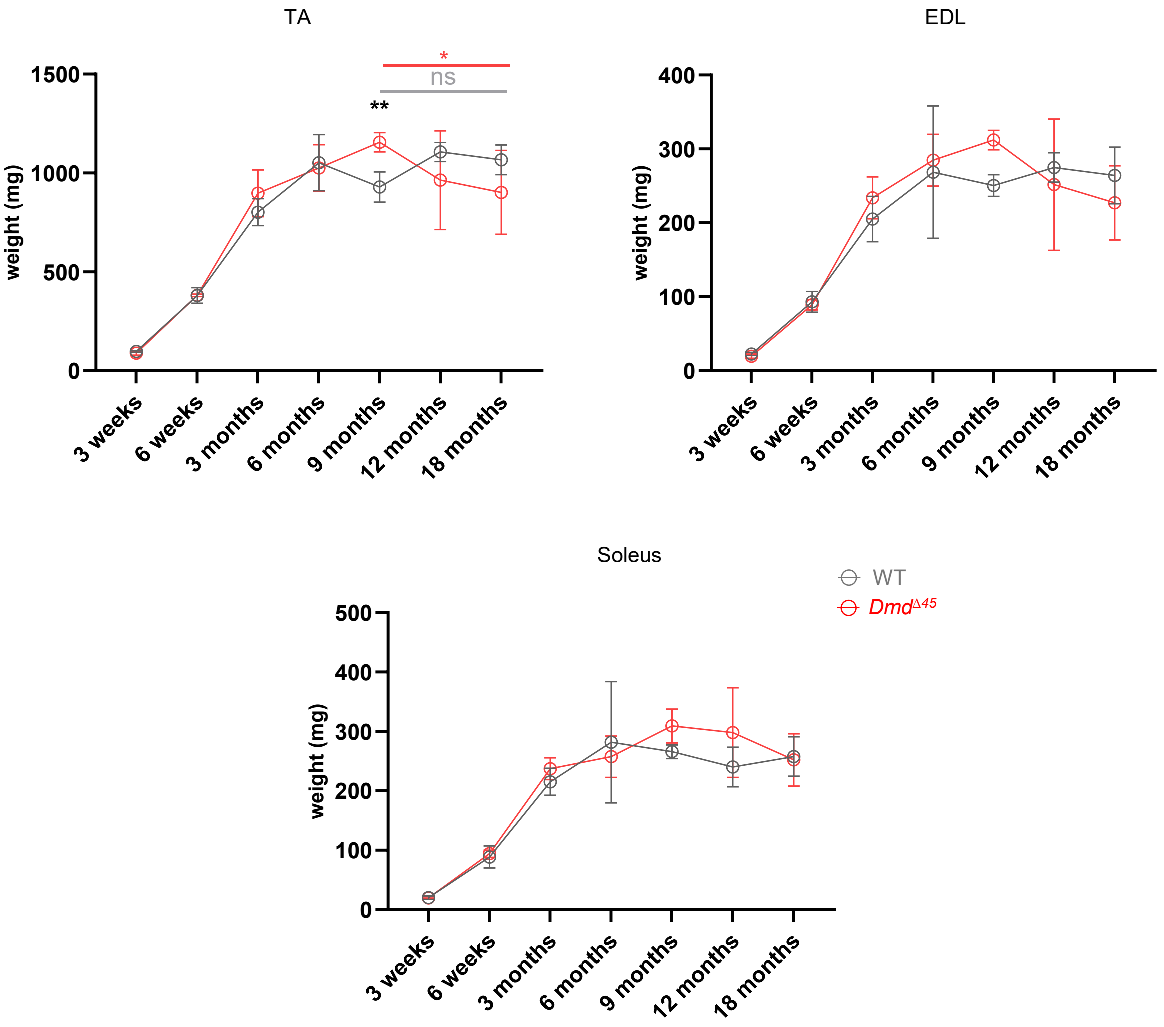

Fig. S2. Muscle weight in WT and DmdΔ45 rats from 3 weeks to 18 months (n = 3–10, mean ± SD). Data were analyzed using two-way ANOVA with genotype and age as factors, followed by Sidak's post hoc multiple comparisons. Significant differences between genotypes at the same age are indicated by black asterisks, while significant differences across ages within the same genotype are indicated in genotype-matched colors (gray for WT, red for *Dmd*$^{\Delta 45}$). ns: not significant; *$p < 0.05$, **$p < 0.01$, ***$p < 0.001$, ****$p < 0.0001$.

Disease Models & Mechanisms • Supplementary information

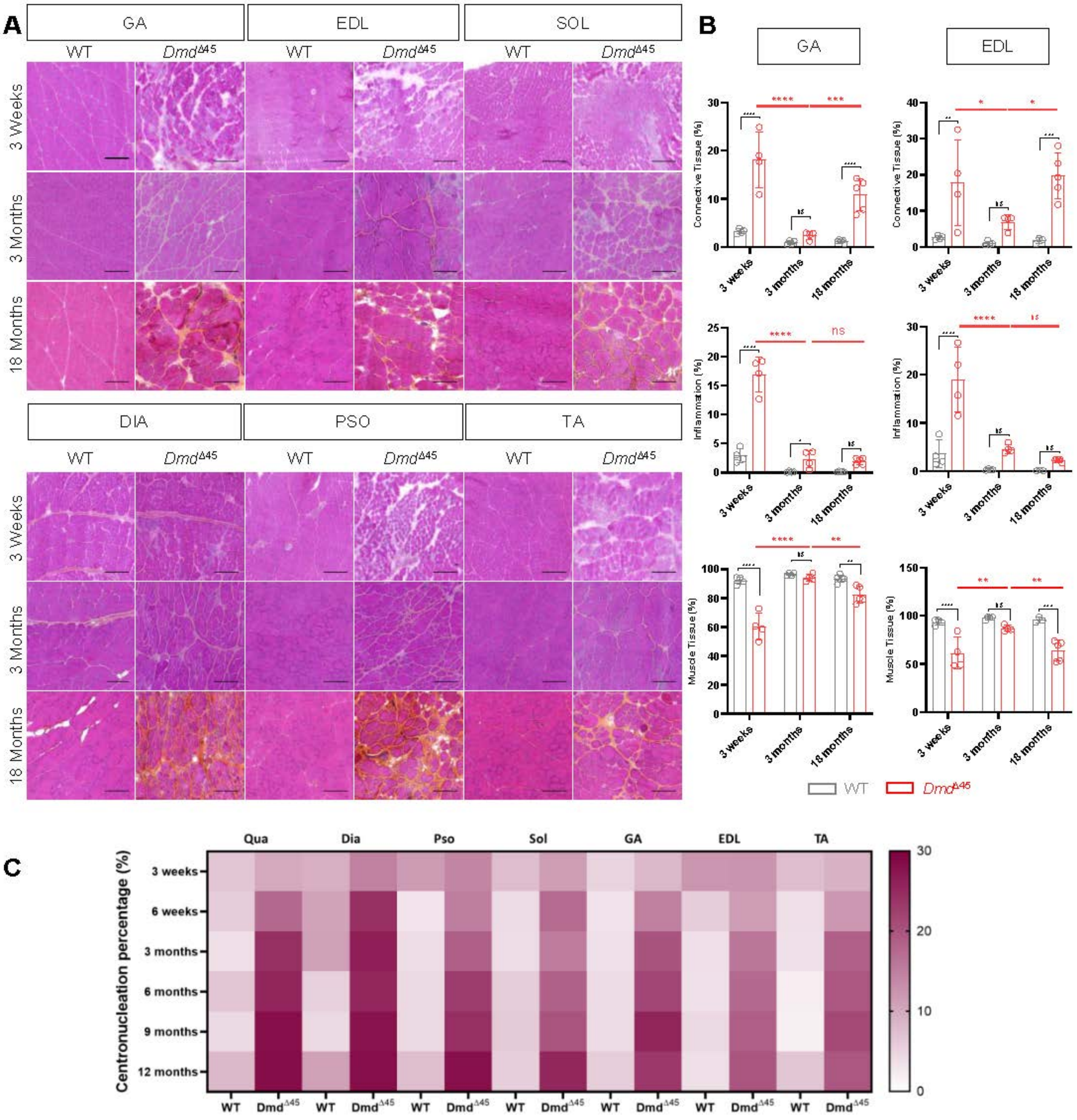

Fig. S3. Histological evaluation of skeletal muscles. (**A**) Representative images of skeletal muscles, Gastrocnemius (GA), EDL, Soleus (SOL), Diaphragm (DIA), Psoas (PSO) and tibialis anterior (TA) cross-sections stained with Hematoxylin Phloxine Saffron (HPS) from WT and *Dmd*^{Δ45} rats at 3 weeks, 3 months and 18 months. Size bar = 200 µm. (**B**) Quantification of connective tissue, inflammation and muscle tissue percentages from HPS-stained cross-sections in GA and EDL (n = 3–5, mean ± SD). (**C**) Heatmap showing centronucleation percentage in different skeletal muscles of WT and *Dmd*^{Δ45} across ages. Two-way ANOVA was performed with factors genotype and age, followed by Sidak's multiple comparisons. Significant differences between genotypes at the same age are indicated with black asterisks, while significant differences across ages within *Dmd*^{Δ45} rats are shown in red. ANOVA p-values, ns: non-singificant, *p < 0.05, **p<0.01, ***p<0.001, ***p=0.0001.

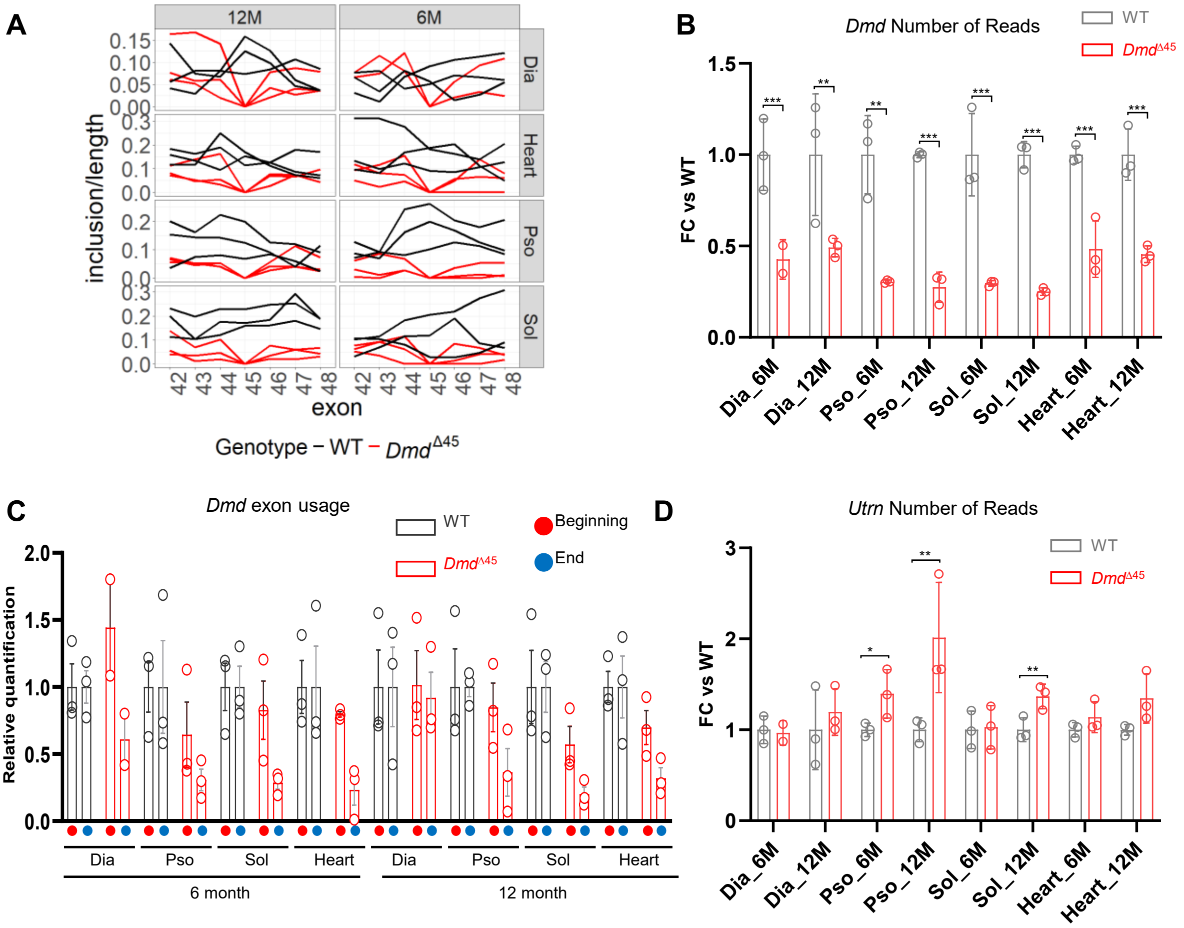

Fig. S4. RNA-seq analysis revealed dysregulated pathways associated to DMD. (**A**) Relative read counts for exons 42 to 48 of the *Dmd* gene in Dia, Pso, Sol, and heart tissues at 6 and 12M. (**B**) Relative read counts for *Dmd* in Dia, Pso, Sol and Heart samples at 6M or 12M (n=3, mean ± SD). (**C**) Relative total exon read counts for *Dmd* in Dia, Pso, Sol and Heart samples at 6M or 12M. The Exons were grouped into two regions: Beginning (exons upstream of exon 45) and End (exon downstream of exon 45) (n=3, mean ± SD). (**D**) Relative read counts of Utrophin (*Utrn*) of the Dia, Pso, Sol and Heart samples at 6M or 12M (n=3, mean ± SD). An unpaired two-tailed t.test was used for statistical comparisons. T.test p-value $^*p < 0.05$, $^{**}p<0.01$, $^{***}p<0.001$.

Disease Models & Mechanisms • Supplementary information

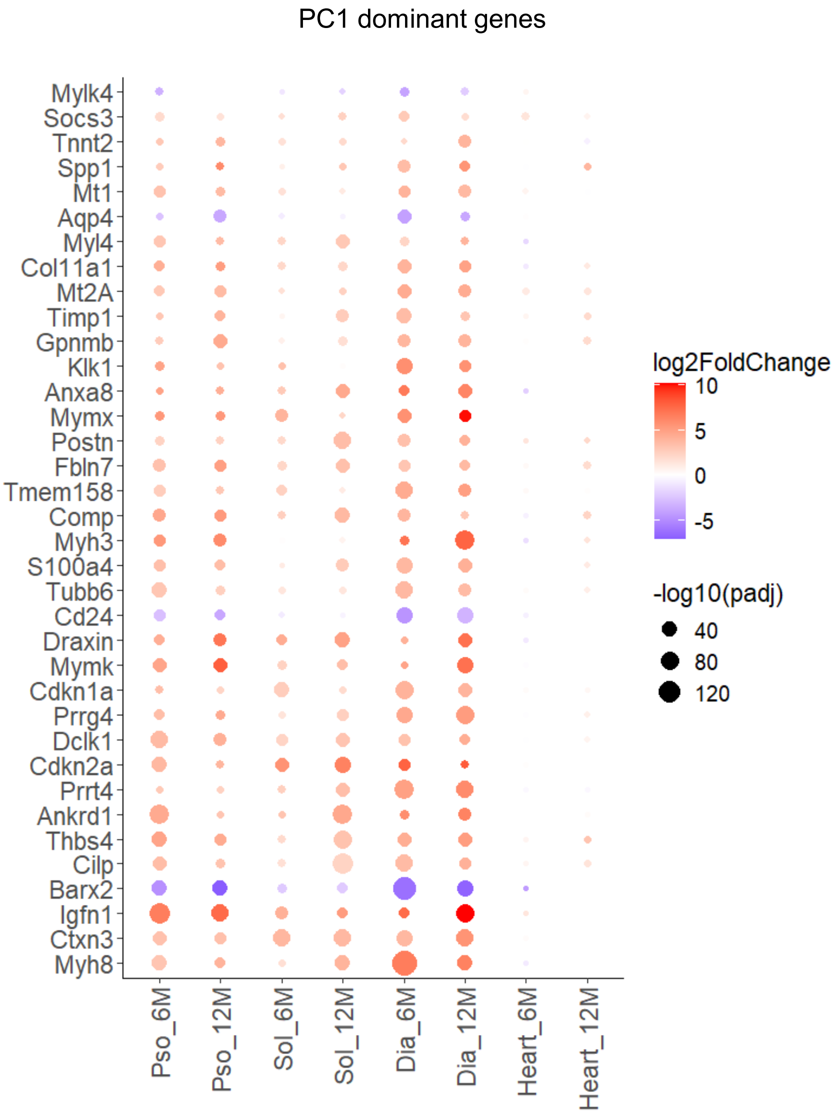

Fig. S5. Dot graph of the PC1 top-contributing genes. Each dot represents a gene in a specific *Dmd*$^{\Delta 45}$ sample. Colour indicates the $\log_2$ fold change, with blue for downregulation and red for upregulation. Dot size reflects the -log10(padj) for that gene in the corresponding *Dmd*$^{\Delta 45}$ sample. Genes are ordered based on the geometric mean of their adjusted p-values across all conditions, highlighting the most consistently significant genes. The result showed that the heart does not present similar dysregulation than in skeletal muscles.

Disease Models & Mechanisms • Supplementary information

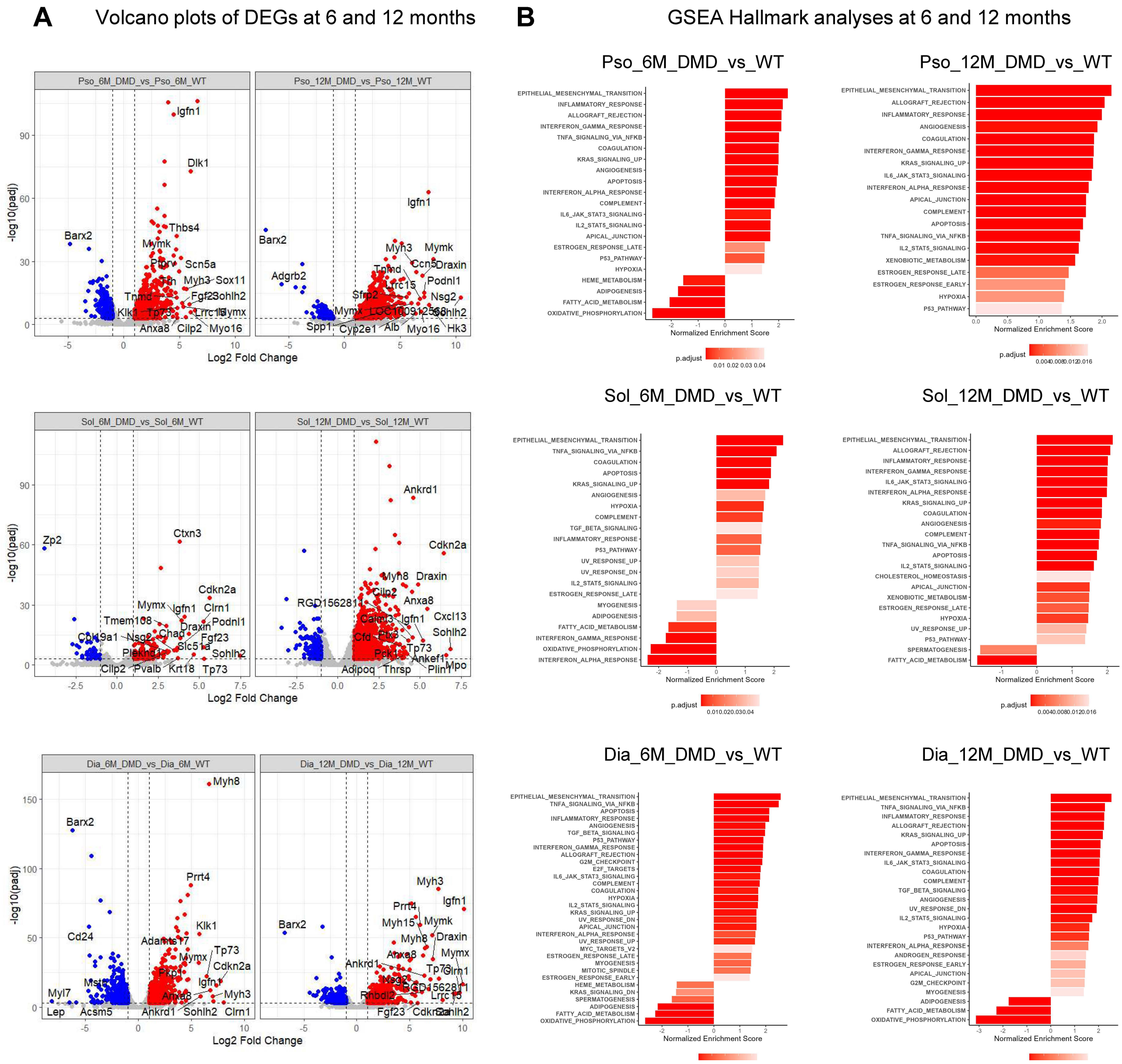

Fig. S6. Differential gene expression and pathway enrichment in dystrophic and WT rat muscles. (**A**) Volcano plots showing differential gene expression in Pso, Sol and Dia muscles, comparing *Dmd*$^{\Delta 45}$ and WT rats at 6 months (left panel) and 12 months (right panel). (**B**) Bar plot summarizing GSEA of Hallmark pathways in Pso, Sol and Dia muscles.

Disease Models & Mechanisms • Supplementary information

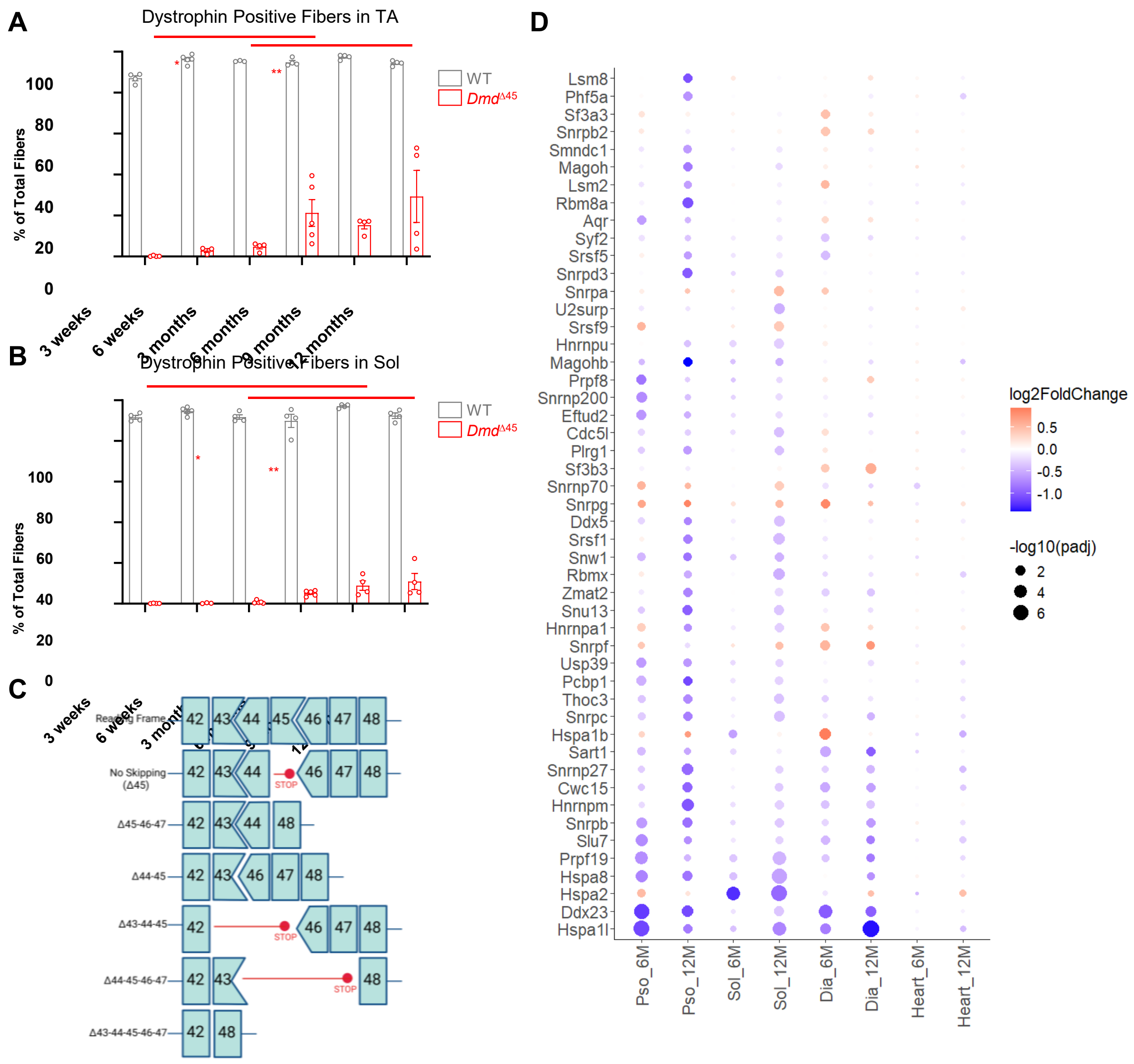

Fig. S7. Partial restoration of dystrophin expression and dysregulation of spliceosome related genes in *Dmd*$^{\Delta 45}$ rats. (**A–B**) Quantification of dystrophin-positive fibers in TA and soleus muscles from 3 weeks to 12 months of age (n = 3–5, mean ± SD). (**C**) Schematic representation of the reading frame across exons 43–48. The native *Dmd*$^{\Delta 45}$ allele leads to a frameshift and premature stop codon (indicated). TOPO cloning identified multiple alternative isoforms generated by exon skipping in different skeletal muscles. Some isoforms (e.g., skipping of exon 44 in addition to exon 45) restore the reading frame and allow expression of a truncated dystrophin, whereas others introduce premature stop codons. (**D**) Dot plot of dysregulated spliceosome-related genes. Each dot represents one gene in a specific DMD sample. Color indicates $\log_2$ fold change (blue, downregulated; red, upregulated), while dot size reflects $-\log_{10}$(padj). Genes are ranked by the geometric mean of their adjusted p-values across all conditions. Two-way ANOVA was performed with factors genotype and age, followed by Sidak's multiple comparisons, for analysis of dystrophin positive fibers. Significant differences across ages within *Dmd*$^{\Delta 45}$ rats are shown in red. ANOVA p-values, *p < 0.05, **p<0.01.

Disease Models & Mechanisms • Supplementary information

Table S1. Primers used for off-target site analysis.

**Off Target Site**	**Exon/Intron/ Intergenic**	**Mismatch to target**	**Primers**	**PCR Product size (bp**
Shroom2	exon	**4**	F : 5'- ATGCCTTCGTGACTCCACAAT -3'<br>R : 5'- CTTGCCAGATCAGACCCACG -3'	307
Wfdc2	exon	**4**	F : 5'- ATGCACACTCTGTAAGGAAGGG -3'<br>R : 5'- CACTTCCCATGGAAAAAGGCG -3'	337
Misfa1	exon	**4**	F : 5'- GATCTTGGCAAAGGAGCGAAC -3'<br>R : 5'- TGCCCCCTGGTGTAAGTCAA -3'	299
Dmrtc1b	exon	**4**	F : 5'- GAGGAGCTGGGCACTTAGAG -3'<br>R : 5'- AGGTCCCAGGTCCAACTACA -3'	324

Table S2. Transthoracic echocardiography parameters. Doppler image analyses were performed only in 3- and 18 months old rat. Significant p-values are indicated in red (mean ± SD). An unpaired two-tailed t.test was used for statistical comparisons. T.test p-value *p < 0.05, **p<0.01, ***p<0.001.

Parameter			Unit	3 Months			6 Months			9 Months			18 Months		
				**WT** (n=8)	***Dmd*$^{\Delta45}$** (n=8)	*p-value*	**WT** (n=5)	***Dmd*$^{\Delta45}$** (n=5)	*p-value*	**WT** (n=7)	***Dmd*$^{\Delta45}$** (n=7)	*p-value*	**WT** (n=5)	***Dmd*$^{\Delta45}$** (n=6)	*p-value*
**General Parameters**		Body Weight	g	497.05 ± 59.49	436.63 ± 48.53	0.04 *	798.4 ± 126.24	500.4 ± 39.42	0.001 **	687.24 ± 22	561.6 ± 20.30	0.001 **	788.6 ± 121	541.7 ± 126.4	0.0094 **
		Heart Rate	bpm	357.75 ± 21.19	352.86 ± 54.66	0.82	349.41 ± 36.22	329.84 ± 26.58	0.36	322.86 ± 39.28	357.14 ± 35.02	0.18	306.48 ± 30.90	307.65 ± 19.91	0.,94
**Left Ventricle (LV) Parameters**	**LV Structural Dimensions**	LV End-Diastolic Diameter (LVEDD)	mm	8.47 ± 0.55	8.72 ± 1.31	0.63	9.18 ± 0.75	8.7 ± 0.91	0.39	8.50 ± 0.59	8.35 ± 0.81	0.68	8.85 ± 0.51	8.84 ± 0.55	0.96
		LV End Diastolic Diameter (LVEDD)	mm	5.23 ± 0.70	5.81 ± 1.12	0.23	5.00 ± 0.29	5.02 ± 0.74	0.96	5.19 ± 0.31	4.83 ± 0.84	0.3	3.76 ± 0.47	5.43 ± 0.91	0.005 **
		LV Anterior Wall Thickness (Diastole)	mm	1.36 ± 0.12	1.47 ± 0.16	0.13	1.84 ± 0.14	1.96 ± 0.15	0.21	1.52 ± 0.23	1.41 ± 0.20	0.35	2.19 ± 0.13	2.11 ± 0.19	0.44
		LV Anterior Wall Thickness (Systole)	mm	1.95 ± 0.28	2.00 ± 0.27	0.74	3.38 ± 0.34	3.22 ± 0.34	0.49	2.02 ± 0.18	2.21 ± 0.30	0.16	4.22 ± 0.16	3.21 ± 0,30	7.67E-05 ***
		LV Posterior Wall Thickness (Diastole)	mm	1.93 ± 0.24	1.76 ± 0.24	0.17	2.09 ± 0.32	2.17 ± 0.20	0.63	1.95 ± 0.24	2.06 ± 0.12	0.46	2,13 ± 0,21	1.93 ± 0.29	0.23
		LV Posterior Wall Thickness (Systole)	mm	3.03 ± 0.31	2.54 ± 0.50	0.03*	3.38 ± 0.25	3.15 ± 0.47	0.37	2.96 ± 0.49	3.09 ± 0.56	0.67	3.73 ± 0.41	2.97 ± 0.40	0.012 *
		LV Mass (Anterior Wall)	mg	1158.9 ± 169.04	1127.16 ± 188.09	0.74	1549.4 ± 111.55	1551.77 ± 378.74	0.99	1158.9 ± 169.04	1127.16 ± 188.09	0.75	1687.95 ± 264.57	1520.30 ± 125.40	0.20
		LV Mass (Corrected)	mg	927.12 ± 135.23	901.73 ± 150.47	0.74	1239.2 3 ± 89.24	1241.41 ± 303	0.99	927.12 ± 135.23	901.73 ± 160.47	0.75	1350.36 ± 211.65	1216.24 ± 100.32	0.20
	**LV Volumes and Function**	End-Diastolic Volume (EDV)	mL	392. 67 ± 58.55	427.35 ± 145.90	0.54	470.72 ± 80.33	420.37 ± 95.77	0.39	396.8 ± 59.70	382.64 ± 78.01	0.2	434.14 ± 55.75	432.62 ± 62.42	0.95
		End-Systolic Volume (ESV)	mL	134.20 ± 40.36	175.11 ± 79.80	0.21	119.16 ± 15.49	122.94 ± 39.60	0.85	129.85 ± 18.14	112.94 ± 47.28	0.47	62.76 ± 16.68	148.44 ± 62.07	0.0156 *
		Stroke Volume (SV)	µL	432.58 ± 138.63	454.38 ± 248.26	0.83	351.56 ± 76.20	297.43 ± 82.90	0.31	478.99 ± 76.49	333.60 ± 112.26	0.025 *	371.37 ± 46.35	284.18 ± 31.12	0.0047 **
		Cardiac Output (CO)	mL/min	156.59 ± 61.28	160.05 ± 86.62	0.93	124.03 ± 33.92	97.01 ± 22.75	0.18	156.59 ± 29.79	119.52 ± 45.11	0.12	114.05 ± 20.43	86.78 ± 9,56	0.0167 *
		Ejection Fraction (EF)	%	66.02 ± 8.41	60.02 ± 2.09	0.12	74.13 ± 5.22	70.51 ± 8.13	0.43	66.95 ± 1.74	70.91 ± 3.1	0.29	85.70 ± 3.30	66.36 ± 9.17	0.0016 **
		Fractional Shortening (FS)	%	38.26 ± 2.34	33.54 ± 1.50	0.11	45.27 ± 4.84	42.25 ± 7.23	0.46	38.77 ± 1.40	42.29 ± 2.59	0.26	57.66 ± 4.38	38.79 ± 7.08	0.00058 ***
		Non-Filling Time (NFT)	ms	110.41 ± 6.92	106.67 ± 7.32	0.31				119.76 ± 7.38	131.59 ± 9.60	0.02 *			
	**LV Time Intervals**	Isovolumetric Contraction Time (IVCT)	ms	30.41 ± 9.07	25 ± 4.24	0.15				33.41 ± 3.96	35.24 ± 7.50	0.58			
		Isovolumetric Relaxation Time (IVRT)	ms	32.78 ± 7.30	29.86 ± 3.53	0.7				35 ± 6.78	46.03 ± 13.86	0.08			
		LV Myocardial Performance Index (MPI)	-	1.18 ± 0.35	0.93 ± 0.17	0.09				1.18 ± 0.19	1.17 ± 0.22	0.91			
		LV MPI Non-Filling Time (MPI NFT)	-	1.16 ± 0.35	0.80 ± 0.2	0.06				1.06 ± 0.25	1.15 ± 0.25	0.49			
**Aortic Parameters**	**Structure**	Aortic Root Diameter	mm	3.38 ± 0.19	3.38 ± 0.47	1	62.78 ± 12.03	62.78 ± 10.75	1	3.64 ± 0.32	3.55 ± 0.32	0.62	3.52 ± 0.16	3.33 ± 0.37	0.32
	**Doppler Measurements**	Aortic Velocity Time Integral (VTI)	mm	47.99 ± 14.31	47.16 ± 12.77	0.9				46.37 ± 8	32.71 ± 6.74	0.0095 **			
		Aortic Mean Velocity	mm/s	374.30 ± 99.80	358.61 ± 70.12	0.72				386.31 ± 63.59	250.01 ± 28.65	0.00073 ***			
		Aortic Peak Velocity	mm/s	793.15 ± 186.63	773.44 ± 165.50	0.83				854.09 ± 174.70	540.88 ± 131.13	0.0056 **			
		Aortic Mean Pressure Gradient	mmHg	0.60 ± 0.3	0.53 ± 0.21	0.63				0.61 ± 0.61	0.26 ± 0.06	0.0022 **			

Disease Models & Mechanisms • Supplementary information

		Aortic Peak Pressure Gradient	mmHg	2.64 ± 1.25	2.49 ± 1.1	0.8		3.03 ± 1.24	1.23 ± 0.60	0.0095 **
		Aortic Peak Pressure	mmHg	2.65 ± 1.10	2.48 ± 1.05	0.75		2.23 ± 0.48	1.32 ± 0.59	0.015 *
		Aortic Ejection Time	ms	54.58 ± 15.70	59.72 ± 5.93	0.4		59.36 ± 11.16	61.70 ± 6.82	0.65
**Mitral Valve Doppler Measurements**		Mitral Valve E Wave Velocity	mm/s	875.13 ± 98.26	884.92 ± 230.39	0.91		839.56 ± 202.11	823.80 ± 92.06	0.85
		Mitral Valve A Wave Velocity	mm/s	640.10 ± 166.55	652.32 ± 217.86	0.9		516.94 ± 83.55	490.55 ± 157.54	0.7
		E/A Ratio	-	1.45 ± 0.41	1.42 ± 0.33	0.88		1.65 ± 0.43	1.8 ± 0.50	0.55
		E' / A' (Tissue Doppler)	-	0.87 ± 0.12	0.83 ± 0.32	0.73		0.87 ± 0.09	0.70 ± 0.21	0.06
		A' / E' (Tissue Doppler)	-	1.17 ± 0.18	1.42 ± 0.63	0.3		1.16 ± 0.12	1.57 ± 0.56	0.085
		E/E' Ratio	-	-18.60 ± 4.23	-23.27 ± 13.11	0.35		-19.21 ± 5.81	-21.87 ± 6.39	0.43
**Pulmonary Artery and Valve Parameters**		Pulmonary Artery Velocity Time Integral (PA VTI)	mm	41.92 ± 5.34	61.22 ± 11.13	0.000579 ***		43.92 ± 3.04	46.22 ± 6.19	0.55
		Pulmonary Artery Mean Velocity	mm/s	412.82 ± 71.03	501.86 ± 110.03	0.075		395.34 ± 45.41	391.71 ± 75.99	0.92
		Pulmonary Artery Peak Velocity	mm/s	782.25 ± 190.19	928.36 ± 132.87	0.096		733.42 ± 104.1	697.60 ± 77.88	0.48
		Pulmonary Artery Mean Gradient	mmHg	0.6975 ± 0.27	1.05 ± 0.508	0.1		0.63 ± 0.14	0.63 ± 0.25	0.99
		Pulmonary Artery Peak Pressure Gradient	mmHg	2.5575 ± 1.44	3.51 ± 1.03	0.155		2.19 ± 0.62	1.97 ± 0.44	0.44
		Pulmonary Valve Peak Velocity	mm/s	747.14 ± 75.66	950.32 ± 131.89	0.002 **		743.03 ± 103.56	706.71 ± 99.64	0.52
		Pulmonary Valve Peak Pressure	mmHg	2.25 ± 0.46	3.67 ± 1.04	0.0032 **		2.25 ± 0.62	2.03 ± 0.57	0.51
		Pulmonary Artery Acceleration Time (PAT)	ms	43.89 ± 6.95	46.74 ± 6.02	0.396		47.46 ± 10.1	43.65 ± 3.25	0.36
		Pulmonary Artery Ejection Time (PET)	ms	109.72 ± 24.72	146.60 ± 19.29	0.0049 **		131.75 ± 38.87	117.14 ± 15.34	0.37
		Pulmonary Artery Hypertension Indicator (PAT/PET)	-	0.42 ± 0.1	0.325 ± 0.07	0.05		0.38 ± 0.08	0.38 ± 0.06	0.91
		Mean Pulmonary Artery Pressure (mPAP)	mmHg	59.25 ± 3.13	57.97 ± 2.7	0.39		57.64 ± 4.54	59.36 ± 1.46	0.36
**Tricuspid Valve Doppler measurements**		Tricuspid Valve Velocity Time Integral (TV VTI)	mm	50.28 ± 15.57	43.56 ± 12.46	0.36		51.61 ± 12.86	43.5 ± 5.67	0.15
		Tricuspid Valve Mean Velocity	mm/s	362.78 ± 88.37	311.19 ± 57	0.19		381.01 ± 71.31	270.81 ± 25.59	0.0023 **
		Tricuspid Valve Peak Velocity	mm/s	740.26 ± 213.41	731.73 ± 116.14	0.92		826.42 ± 154.24	657.28 ± 76.01	0.023 *
		Tricuspid Valve Mean Pressure Gradient	mmHg	0.56 ± 0.23	0.40 ± 0.15	0.11		0.60 ± 0.24	0.30 ± 0.06	0.008 **
		Tricuspid Valve Peak Pressure Gradient	mmHg	2.37 ± 1.09	2.19 ± 0.69	0.7		2.81 ± 1.10	1.76 ± 0.39	0.035 *
		Tricuspid Valve Peak Pressure	mmHg	2.35 ± 1.12	2.19 ± 0.69	0.73		2.81 ± 1.10	1.75 ± 0.40	0.033 *
		Tricuspid Valve E Wave Velocity	mm/s	426.33 ± 104	407.29 ± 68.06	0.67		468.47 ± 58.02	368.09 ± 53.11	0.0055 **
		Tricuspid Valve A Wave Velocity	mm/s	734.36 ± 192.51	778.38 ± 102.76	0.58		884.28 ± 171.97	674.31 ± 84.67	0.013 *
		Tricuspid Valve E/A Ratio	-	0.59 ± 0.08	0.53 ± 0.07	0.1		0.54 ± 0.06	0.55 ± 0.07	0.77

Disease Models & Mechanisms • Supplementary information

Table S3. Top contributing genes to PC1 from PCA analysis. Genes with an average contribution score >0.03 were selected and ranked by absolute contribution to the first principal component (PC1). The table separates upregulated and downregulated genes. For each gene, the full gene name is provided along with references reporting its dysregulation in Duchenne muscular dystrophy (DMD), when available.

Upregulated genes

gene	Full name	Reference in DMD
Ankrd1	Ankyrin Repeat Domain 1	(Nakada et al., 2003)
Igfn1	Ig Like And Fibronectin Type III Domain Containing 1	-
Myh3	Myosin Heavy Chain 3	(Kimura et al., 2024)
Myh8	Myosin Heavy Chain 8	(Hildyard et al., 2022)
Thbs4	Thrombospondin 4	(Heezen et al., 2023)
Cdkn1a	Cyclin Dependent Kinase Inhibitor 1A	(Young et al., 2021)
Comp	Cartilage Oligomeric Matrix Protein	(Taglietti et al., 2022)
S100a4	S100 Calcium Binding Protein A4	-
Cilp	Cartilage Intermediate Layer Protein	-
Anxa8	Annexin A8	-
Postn	Periostin	(Marotta et al., 2009)
Dclk1	Doublecortin Like Kinase 1	-
Cdkn2a	Cyclin Dependent Kinase Inhibitor 2A	(Sugihara et al., 2020)
Mt2A	Metallothionein 2A	-
Draxin	Dorsal Inhibitory Axon Guidance Protein	-
Mymk	Myomaker, Myoblast Fusion Factor	(Gosselin et al., 2022)
Prrt4	Proline Rich Transmembrane Protein 4	-
Ctxn3	Cortexin 3	-
Timp1	TIMP Metallopeptidase Inhibitor 1	(von Moers et al., 2005)
Mymx	Myomixer, Myoblast Fusion Factor	(Gosselin et al., 2022)
Gpnmb	Glycoprotein Nmb	(Patsalos et al., 2024)
Prrg4	Proline Rich And Gla Domain 4	-
Tubb6	Tubulin Beta 6 Class V	(Randazzo et al., 2019)
Spp1	Secreted Phosphoprotein 1/ Osteopontin	(Porter et al., 2002)
Myl4	Myosin Light Chain 4	(Heezen et al., 2023)
Klk1	Kallikrein 1	-
Mt1a	Metallothionein 1A	-
Tmem158	Transmembrane Protein 158	-
Tnnt2	Troponin T2, Cardiac Type	(Bakay et al., 2002; Xin and Liu, 2025)
Fbln7	Fibulin 7	-
Cd24	CD24 Molecule	-
Col11a1	Collagen Type XI Alpha 1 Chain	-
Socs3	Suppressor of cytokine signalling 3	-

Downregulated genes

gene	Full name	Reference in DMD
Barx2	BARX Homeobox 2	-
Mylk4	Myosin Light Chain Kinase Family Member 4	-
Aqp4	Aquaporin 4	(Wakayama et al., 2002)

Disease Models & Mechanisms • Supplementary information

Table S4. Commonly dysregulated genes among skeletal muscles. The genes are ranked by the average log_2 fold change (FC) across samples. The 20 genes with the highest average fold change were selected out of 26 genes for down-regulated genes at 6 months, 179 genes for up-regulated genes at 6 months, 58 genes for down-regulated genes at 6 months and 625 genes for up-regulated genes at 12 months. Genes that are found at the 2 time-points are in red. Genes that are found in the top contribution PC1 list are highlighted in yellow. The *Dmd* gene is in bold.

Up_6M	FC	Up_12M	FC	Down_6M	FC	Down_12M	FC
Sohlh2	6.80	*Sohlh2*	8.84	*Barx2*	-4.47	*Barx2*	-5.41
Igfn1	6.03	*Igfn1*	7.57	*Zp2*	-3.23	*Unc5a*	-3.53
Clrn1	5.76	*Draxin*	6.33	*Oxtr*	-3.16	*AABR07021734.1*	-3.51
Cdkn2a	5.69	*Lrrc15*	6.20	*AABR07021734.1*	-2.96	*Slc6a2*	-3.07
Pilrb-ps6	5.63	*Th*	6.20	*Asb4*	-2.69	*Asb4*	-3.05
Serpina3c	5.48	*Mymk*	6.17	*Adra2c*	-2.48	*Nppc*	-2.88
Tp73	5.48	*Cdkn2a*	6.12	*Adig*	-2.43	*Gm37419*	-2.41
Mymx	4.98	*Clec2dl1*	6.02	*Dpep1*	-2.18	*Mogat2*	-2.33
Fgf23	4.94	*Nsg2*	5.84	*Ppp1r1a*	-2.13	*Cacng7*	-2.32
Anxa8	4.78	*Bin2a*	5.59	*Rhou*	-2.08	*LOC102551557*	-2.26
Klk1	4.60	*LOC120103148*	5.57	*Cacng7*	-2.03	*Gucy2g*	-1.98
Gbx2	4.58	*Tnn*	5.57	*Inpp5j*	-1.84	*Rnf43*	-1.97
Serpinb2	4.49	*Otog*	5.51	*Fbxo44*	-1.82	*Ppp1r1a*	-1.94
Ankrd1	4.46	*Klra5*	5.38	*Smtnl2*	-1.69	*Spdya*	-1.94
Lrrc15	4.37	*Ptx4*	5.21	*PCOLCE2*	-1.62	*Rhou*	-1.92
Tmem184a	4.23	*Bicdl2*	5.18	*LOC120098897*	-1.61	*P3r3urf*	-1.86
Draxin	4.22	*Podnl1*	5.13	***Dmd***	-1.59	*Nrep*	-1.84
Ptx4	4.22	*RGD1562811*	5.09	*Trim45*	-1.58	*Pcdhga1*	-1.73
Rassf7	4.21	*Tnmd*	5.07	*Ppm1j*	-1.56	*AABR07052523.2*	-1.71
Clec2dl1	4.16	*Tmem184a*	5.04	*Rab3b*	-1.47	*Tuba8*	-1.69

Table S5. Additional dysregulated genes in both Pso and Sol. The genes are ranked by the average $\log_2$ fold change across samples. When more than 20 genes were identified, those with the highest average fold change were selected with 20 out of 34 for the down regulated genes at 6M, 20 out of 56 for the upregulated genes at 6 months and 20 out of 797 at 12 months. Genes that are found at the 2 time-points are in red. Genes that are found in the top contribution PC1 list are highlighted in yellow.

Up_6M	FC	Up_12M_	FC	Down_6M	FC	Down_12M	FC
Yjefn3	4.02	AABR07065812.2	6.96	Akr1c3	-2.56	Rpl10l	-2.40
Crocc2	3.61	Steap1	6.02	Caly	-2.27	Asb9	-2.39
Tmem108	3.39	Alb	5.86	Gucy2g	-1.96	Sim1	-1.94
Nexmif	3.11	Mpo	5.70	Ntmt2	-1.83	Fzd10	-1.85
Cfap20dc	2.65	Col10a1	5.34	Tnfrsf11b	-1.50	Crb1	-1.84
Vwa2	2.62	AABR07053830.1	4.80	Epop	-1.39	Ddah1	-1.66
S100a3	2.54	Ankef1	4.50	Bbs9	-1.22	Mt-nd4l	-1.61
Piezo2	2.52	Pwwp4	4.48			Homer1	-1.60
Il22ra2	2.48	Tspoap1	4.47			Clcn1	-1.58
Dhrs9	2.41	Klhdc7a	4.45			Mir3582	-1.52
C3h9orf50	2.34	Cfd	4.41			Crppa	-1.49
Pamr1	2.30	Cyp2e1	4.37			Zfp770	-1.46
Ism1	2.25	Slfn4	4.35			Ehbp1	-1.38
Glt6d1	2.24	C4a	4.30			Gen1	-1.38
Tnnt2	2.23	Plin1	4.24			Eya1	-1.36
Krt8	2.16	Fcrl1	4.21			Carnmt1	-1.32
Cdhr4	2.11	Lgals12	4.18			Iqub	-1.32
Musk	2.05	Retn	4.13			LOC120093538	-1.25
Ociad2	2.05	Blk	4.05			Plaat1	-1.25
Sfrp2	2.01	Rhbg	4.02			Epop	-1.24

Disease Models & Mechanisms • Supplementary information

Table S6. Commonly dysregulated genes in heart. The genes are ranked by the log_2 fold change (FC) across samples. Only the top 20 first genes are shown. Genes that are found at the 2 time points are in red. The *dmd* gene is in bold.

6M UP		12M UP		6M DN		12M DN	
**Gene**	**FC**	**Gene**	**FC**	**Gene**	**FC**	**Gene**	**FC**
Pilrb-ps6	4.41	Spp1	3.81	LOC100912568	-7.47	Tnnc2	-4.60
Fgf23	4.31	Kcne1	3.60	Cacna1s	-3.67	Cacna1s	-3.97
Cxcl2	3.28	Clec2dl1	3.36	AABR07021734.1	-3.63	Actn3	-3.86
Egr1	3.25	Mmp12	3.15	Adh6	-3.35	Atp2a1	-3.78
Hist1h4m	3.09	Thbs4	3.12	Zp2	-3.19	Pvalb	-3.41
Fos	3.00	Ltbp2	2.84	Slc17a7	-2.65	Tnni2	-3.08
Nr4a1	2.44	Olr1	2.75	RT1-CE16	-2.47	Slc4a1	-3.03
Cxcl1	2.37	Trh	2.65	Olr35	-2.39	Myh2	-2.90
Egr3	2.35	Thbs1	2.61	Rpl39	-2.10	Smtnl1	-2.84
Btg2	2.10	Nefm	2.58	Lrrn2	-1.82	Myl11	-2.81
Arc	2.08	Rgs1	2.43	Fam222a	-1.72	Mylk2	-2.73
Egr2	2.06	Serpina3a	2.36	Neb	-1.67	Ryr1	-2.58
Ier2	2.03	Nrg1	2.28	Alpg	-1.55	Slc9a2	-2.32
Zfp36	1.91	Ptgs2	2.28	Ano5	-1.41	Fam131c	-2.09
Rgs1	1.84	Comp	2.27	Pxmp4	-1.41	Slc17a7	-2.04
Ccn1	1.80	Fhad1	2.26	**Dmd**	-1.35	Myom3	-1.90
Pygl	1.74	Tnfrsf11b	2.18	Csdc2	-1.34	Atp6ap1l	-1.79
Nr4a2	1.67	Piezo2	2.16	AY172581.19	-1.33	Pnpla3	-1.75
Thbs1	1.62	Crtac1	2.15	Atp1a3	-1.28	H2ac1	-1.73
Nfkbiz	1.51	Olfm2	2.14	Susd5	-1.25	Crybb1	-1.69

Disease Models & Mechanisms • Supplementary information

Table S7. Primer sequences

Category	Name	Sequences or epitope
**Genotyping primers**	Forward	5'-TCCTTGTGGGGACAAGAAATCG-3'
	Reverse	5'-ACAGTCTTACTAAGCACAGCTTTC-3'
**Sequencing primer**	FP2	5'-GCTCTTGAGAAGGTTTCCAACTA-3'
**RT-PCR primers**	cDNA_FP42	5'-CGACTGAAGATATGCCTTTGGA-3'
	cDNA_RP48	5'-CTGGCTGACTTGGTTGGTTAT-3'
	cDNA_HumFP42	5'-TCACTCATGTCTCACAAGCCC-3'
	cDNA_HumRP48	5'-GGAGATAACCACAGCAGCAGA-3'

Table S8. Antibodies.

A) Immunohistofluorescence antibodies

Name	Recognised epitope
Dys-b (Leica *NCL-DYSB*)	Dystrophin N-terminal
Dys-2 (Leica *NCL-DYS2*)	Dystrophin C-terminal
Anti-dystrophin (Abcam *ab15277*)	Dystrophin C-terminal
Anti-laminin (Abcam *ab11575*)	Laminin
Anti-utrophin (Leica *DRP2-CE*)	Utrophin N-terminal
Anti-MHCd (Leica *NCL-MHCd*)	MHCd
Alexa fluor 594 Donkey anti-rat IgG Highly Cross-Adsorbed Secondary (Life technologies *A-21209*)	Rat IgG (Heavy and light chains)
Alexa fluor 594 Donkey anti-mouse (Life technologies *A21203*)	Mouse IgG (heavy and light chains)
Alexa fluor 488 Goat anti-rabbit (Life technologies *A11008*)	Rabbit IgG (heavy and light chains)

Disease Models & Mechanisms • Supplementary information

B) Western Blot antibodies

Name	Recognized epitope
Dys-b (Leica *NCL-DYSB*)	Dystrophin N-terminal
Dys-2 (Leica *NCL-DYS2*)	Dystrophin C-terminal
Anti-alpha actinin 4 (Life technologies *PA5-22259*)	Alpha actinin 4 (within amino acids 206 and 542)
IRDye® 800CW Donkey anti-Mouse IgG Secondary Antibody (Li-cor *926-32212*)	Mouse IgG (Heavy and light chains)
IRDye® 680CW Donkey anti-Rabbit IgG Secondary Antibody (Li-cor *926-68073*)	Rabbit IgG (Heavy and light chains)

Dataset 1. List of predicted off-target sites for the sgRNA.

Available for download at
https://journals.biologists.com/dmm/article-lookup/doi/10.1242/dmm.052578#supplementary-data

Dataset 2. List of DEGs from RNA sequencing in skeletal and cardiac muscles at 6 and 12 months.

Available for download at
https://journals.biologists.com/dmm/article-lookup/doi/10.1242/dmm.052578#supplementary-data

Disease Models & Mechanisms • Supplementary information

Disease Models & Mechanisms • Supplementary information

Reference list for Table S3

Bakay, M., Zhao, P., Chen, J., Hoffman, E.P., 2002. A web-accessible complete transcriptome of normal human and DMD muscle. Neuromuscular Disorders, ENMC Special Centennial Workshop Supplement: Therapeutic Possibilities in Duchenne Muscular Dystrophy, Naarden, The Netheralnds, 30 November-2 December 2001 12, S125–S141. https://doi.org/10.1016/S0960-8966(02)00093-7

Gosselin, M.R., Mournetas, V., Borczyk, M., Verma, S., Occhipinti, A., Róg, J., Bozycki, L., Korostynski, M., Robson, S.C., Angione, C., Pinset, C., Gorecki, D.C., 2022. Loss of full-length dystrophin expression results in major cell-autonomous abnormalities in proliferating myoblasts. eLife 11, e75521. https://doi.org/10.7554/eLife.75521

Heezen, L.G.M., Abdelaal, T., van Putten, M., Aartsma-Rus, A., Mahfouz, A., Spitali, P., 2023. Spatial transcriptomics reveal markers of histopathological changes in Duchenne muscular dystrophy mouse models. Nat Commun 14, 4909. https://doi.org/10.1038/s41467-023-40555-9

Hildyard, J.C.W., Riddell, D.O., Harron, R.C.M., Rawson, F., Foster, E.M.A., Massey, C., Taylor-Brown, F., Wells, D.J., Piercy, R.J., 2022. The skeletal muscle phenotype of the DE50-MD dog model of Duchenne muscular dystrophy. Wellcome Open Res 7, 238. https://doi.org/10.12688/wellcomeopenres.18251.1

Kimura, S., Miyake, N., Ozasa, S., Ueno, H., Ohtani, Y., Takaoka, Y., Nishino, I., 2024. Increase in cathepsin K gene expression in Duchenne muscular dystrophy skeletal muscle. Neuropathology 44, 411–421. https://doi.org/10.1111/neup.12995

Marotta, M., Ruiz-Roig, C., Sarria, Y., Peiro, J.L., Nuñez, F., Ceron, J., Munell, F., Roig-Quilis, M., 2009. Muscle genome-wide expression profiling during disease evolution in mdx mice. Physiological Genomics 37, 119–132. https://doi.org/10.1152/physiolgenomics.90370.2008

Nakada, C., Tsukamoto, Y., Oka, A., Nonaka, I., Takeda, S., Sato, K., Mori, S., Ito, H., Moriyama, M., 2003. Cardiac-Restricted Ankyrin-Repeated Protein Is Differentially Induced in Duchenne and Congenital Muscular Dystrophy. Laboratory Investigation 83, 711–719. https://doi.org/10.1097/01.LAB.0000067484.35298.1A

Patsalos, A., Halasz, L., Oleksak, D., Wei, X., Nagy, G., Tzerpos, P., Conrad, T., Hammers, D.W., Sweeney, H.L., Nagy, L., 2024. Spatiotemporal transcriptomic mapping of regenerative inflammation in skeletal muscle reveals a dynamic multilayered tissue architecture. J Clin Invest 134. https://doi.org/10.1172/JCI173858

Porter, J.D., Khanna, S., Kaminski, H.J., Rao, J.S., Merriam, A.P., Richmonds, C.R., Leahy, P., Li, J., Guo, W., Andrade, F.H., 2002. A chronic inflammatory response dominates the skeletal muscle molecular signature in dystrophin-deficient mdx mice. Hum Mol Genet 11, 263–272. https://doi.org/10.1093/hmg/11.3.263

Randazzo, D., Khalique, U., Belanto, J.J., Kenea, A., Talsness, D.M., Olthoff, J.T., Tran, M.D., Zaal, K.J., Pak, K., Pinal-Fernandez, I., Mammen, A.L., Sackett, D., Ervasti, J.M., Ralston, E., 2019. Persistent upregulation of the β-tubulin tubb6, linked to muscle regeneration, is a source of microtubule disorganization in dystrophic muscle. Hum Mol Genet 28, 1117–1135. https://doi.org/10.1093/hmg/ddy418

Sugihara, H., Teramoto, N., Nakamura, K., Shiga, T., Shirakawa, T., Matsuo, M., Ogasawara, M., Nishino, I., Matsuwaki, T., Nishihara, M., Yamanouchi, K., 2020. Cellular senescence-mediated exacerbation of Duchenne muscular dystrophy. Sci Rep 10, 16385. https://doi.org/10.1038/s41598-020-73315-6

Taglietti, V., Kefi, K., Bronisz-Budzyńska, I., Mirciloglu, B., Rodrigues, M., Cardone, N., Coulpier, F., Periou, B., Gentil, C., Goddard, M., Authier, F.-J., Pietri-Rouxel, F., Malfatti, E., Lafuste, P., Tiret, L., Relaix, F., 2022. Duchenne muscular dystrophy trajectory in R-DMDdel52 preclinical rat model identifies COMP as biomarker of

fibrosis. acta neuropathol commun 10, 1–19. https://doi.org/10.1186/s40478-022-01355-2

von Moers, A., Zwirner, A., Reinhold, A., Brückmann, O., van Landeghem, F., Stoltenburg-Didinger, G., Schuppan, D., Herbst, H., Schuelke, M., 2005. Increased mRNA expression of tissue inhibitors of metalloproteinase-1 and -2 in Duchenne muscular dystrophy. Acta Neuropathol 109, 285–293. https://doi.org/10.1007/s00401-004-0941-0

Wakayama, Y., Jimi, T., Inoue, M., Kojima, H., Murahashi, M., Kumagai, T., Yamashita, S., Hara, H., Shibuya, S., 2002. Reduced Aquaporin 4 Expression in the Muscle Plasma Membrane of Patients With Duchenne Muscular Dystrophy. Arch Neurol 59, 431–437. https://doi.org/10.1001/archneur.59.3.431

Xin, J., Liu, S., 2025. Identifying hub genes and dysregulated pathways in Duchenne muscular dystrophy. International Journal of Neuroscience 135, 375–387. https://doi.org/10.1080/00207454.2024.2302551

Young, L.V., Morrison, W., Campbell, C., Moore, E.C., Arsenault, M.G., Dial, A.G., Ng, S., Bellissimo, C.A., Perry, C.G.R., Ljubicic, V., Johnston, A.P., 2021. Loss of dystrophin expression in skeletal muscle is associated with senescence of macrophages and endothelial cells. American Journal of Physiology-Cell Physiology 321, C94–C103. https://doi.org/10.1152/ajpcell.00397.2020
